# Characterization of *Toxoplasma* DegP, a rhoptry serine protease crucial for lethal infection in mice

**DOI:** 10.1371/journal.pone.0189556

**Published:** 2017-12-15

**Authors:** Gaelle Lentini, Hiba El Hajj, Julien Papoin, Gamou Fall, Alexander W. Pfaff, Nadim Tawil, Catherine Braun-Breton, Maryse Lebrun

**Affiliations:** 1 UMR 5235 CNRS, Université de Montpellier, Montpellier, France; 2 Department of Internal Medicine and Experimental Pathology, Immunology and Microbiology, American University of Beirut, Beirut, Lebanon; 3 Institut de Parasitologie et Pathologie Tropicale, EA 7292, Fédération de Médecine Translationnelle, Université de Strasbourg, Strasbourg, France; University of Wisconsin Medical School, UNITED STATES

## Abstract

During the infection process, Apicomplexa discharge their secretory organelles called micronemes, rhoptries and dense granules to sustain host cell invasion, intracellular replication and to modulate host cell pathways and immune responses. Herein, we describe the *Toxoplasma gondii* Deg-like serine protein (*Tg*DegP), a rhoptry protein homologous to High temperature requirement A (HtrA) or Deg-like family of serine proteases. *Tg*DegP undergoes processing in both types I and II strains as most of the rhoptries proteins. We show that genetic disruption of the *degP* gene does not impact the parasite lytic cycle *in vitro* but affects virulence in mice. While in a type I strain DegP^I^ appears dispensable for the establishment of an infection, removal of DegP^II^ in a type II strain dramatically impairs the virulence. Finally, we show that KO-*DegP*^*II*^ parasites kill immunodeficient mice as efficiently as the wild-type strain indicating that the protease might be involved in the complex crosstalk that the parasite engaged with the host immune response. Thus, this study unravels a novel rhoptry protein in *T*. *gondii* important for the establishment of lethal infection.

## Introduction

The protozoan parasite *Toxoplasma gondii* is an obligate intracellular parasite responsible for toxoplasmosis. It belongs to the phylum Apicomplexa, which includes other significant human pathogens such as *Plasmodium falciparum*, the causative agent of malaria. Toxoplasmosis is usually asymptomatic and well controlled by the immune system of immunocompetent individuals. Following a short acute phase, achronic infection is established by the persistence of latent intracellular bradyzoite stages primarily encysted in the central nervous system and in striated muscles. This latency is well recognized as a strategy for efficient transmission and relies on several factors including host responses, parasite replication and genotypes.

*T*. *gondii* strains fall at least in six major clonal lineages which differ by less than 1% at the DNA level [1–4]. However, each strain harbors its own strategy to modulate the host immune signaling pathways resulting in strong phenotypic differences in the laboratory mouse models (for a review see [5,6]). Infections by type I parasites typified by RH strain is fatal (LD_100_ = 1) in most laboratory mouse strains whereas infections with type II such as Prugniaud (Pru) and ME49 (LD_50_ ~10^2^) or type III such as NED (LD_50_~10^3^) strains generally results in lifelong latent infections characterized by dormant tissue cyst formation [7].

The control of host cell protection against *Toxoplasma* requires an active type 1 cytokine response typified by the production of pro-inflammatory cytokines. However, as a pervasive pathogen, *Toxoplasma* has developed strategies to compromise host mechanisms, avoiding parasite killing. Opposite effects in the immune modulation adopted by the parasites are observed between different strains of *Toxoplasma* [5]. For instance, virulent type I strains down regulate the inflammatory response and control the mechanisms of parasite killing, while avirulent type II and III strains have developed strategies to limit infection and premature death of the host to favor transmission [5]. To do so, a fine balance between the parasite replication and the host immune response is required to preserve the host from the immunopathology effect of pro-inflammatory cytokines induced by type II effectors and to sustain moderate type II strains persistence in animals.

The underlying genetic loci responsible for the most striking phenotypic differences between the three main clonal lineages of *Toxoplasma* have been mapped using classical genetics of pairwise genetic crosses between strains [8–12]. Most of the effectors identified so far originate from the secretory organelles, i.e rhoptries and dense granules. In type I strains, proteins of both organelles co-opt to dampen host responses and favor parasite replication and high virulence. In type II strains, those effectors are mostly inactive due to polymorphism and/or differential expression in a way that control parasite dissemination and persistence in the host. Furthermore, type II strains also express dense granule proteins which participate in the modulation of host cell functions [10,13,14].

Here, we describe a rhoptry protein, *Tg*DegP that plays a pivotal contribution to the virulence of *T*. *gondii*. *Tg*DegP is a serine protease belonging to the HtrA (High Temperature Requirement A) or Deg-like family of serine proteases [15]. While DegP is dispensable for the lytic cycle of both RH type I and Pru type II strains, a marked decrease of acute virulence in Swiss mice is observed in *DegP* knock-out type II strain (KO-*DegP*^*II*^). In contrast, KO-*DegP*^*II*^ parasites kill immunodeficient mice as efficiently as the wild-type strain. In conclusion, genetic ablation of *Tg*DegP suggests a role for this serine protease in immune evasion.

## Materials and methods

### Ethics statement

The recommendations of the European Union guidelines for the handling of laboratory animals was followed for this study. Production of DegP and DegP* antibodies via rat and mouse immunizations was conducted at the CRBM animal facility (Montpellier) and approved by the Committee on the Ethics of Animal Experiments (Languedoc-Roussillon, Montpellier) (Permit Number: D34-172-4, delivered on 20/09/2009).

All mice protocols were approved by the Institutional Animal Care and Utilization Committee (IACUC) of the American University of Beirut (IACUC Permit Number IACUC#14-3-295). Mice were housed in dedicate pathogen free facilities and humane endpoints were used as requested by the AUB IACUC according to AAALAC (Association for Assessment and Accreditation of Laboratory Animal Care International) guidelines and guide of animal care use book (Guide, NRC 2011). Mice were monitored on a daily basis. Eye pricks were done following deep anesthesia with isoflurane by inhalation. One eye per animal was pricked allowing to collect 50uL of blood. Mice were sacrificed if any of the following abnormal ethical features are noticed as described previously [16]. Animals were deeply anesthetized before cervical dislocation and no unexpected death was observed.

### Mammalian cells and parasite cultures

Tachyzoites from RH, RHΔ*Ku80* strain or PruΔ*Ku80* (deleted for the *ku80* gene [17,18]) were used throughout this study. Parasites were grown in human foreskin fibroblasts (HFFs) (American Type Culture Collection-CRL 1634) in Dulbecco’s Modified Eagle’s Medium (DMEM) (GIBCO, Invitrogen) supplemented with 5% of fetal calf serum (FCS), 1% penicillin-streptomycin and 1% glutamine. BHK-21 cells (American Type Culture Collection-CCL 10) were grown in BHK-21 medium (Gibco-BRL) supplemented with 5% FCS, 2 mM tryptose, 100 U/ml penicillin and 100 μg/ml streptomycin.

### Transient transfection of mammalian cells and generation of transgenic parasites

For BHK-21, 3×10^5^ BHK-21 cells were grown on coverslips for 24 h in 6-well plate prior transfection using Lipofectamine reagent (Gibco-BRL) as instructed by the manufacturer. Cells were grown for an additional 24 h before western blot analysis (see below). Parasite transfection and selection was performed as previously described [19]. Parasites were selected in presence of mycophenolic acid (20μg/mL) and xanthine (50μg/mL) for HXGPRT selection or pyrimethamine (1μM) for DHFR-TS selection. The selection of revertant parasites was made in the presence of 300μg/ml of 6-thioxanthine and their sensitivity to mycophenolic acid (20μg/mL) and xanthine (50μg/mL) was assessed. The isolation of clonal transgenic populations was performed using limiting dilution in 96-well plates.

### Antibodies

The antibodies used and their dilution for western blot (WB) and immunofluorescence (IFA) were as follows:

- mouse monoclonal antibodies (MAb) T4 1E5 anti-SAG1, 1:2000 (IFA), 1:2000 (WB) [20]
- rabbit anti-ROP1, 1:3000 (IFA) [21]
- rabbit anti-ROP2 1:500 (IFA) [21]
- mouse anti-Ty MAb, 1:200 (WB) [22]
- rat serum anti-DegP, 1:1000 (IFA), 1:1000 (WB) (this study)
- mouse serum anti-DegP* 1:200 (WB) (this study)
- rabbit anti-RON4, 1:500 (IFA) [23]

For IFA studies, the secondary antibodies used were AlexaFluor 488 (Sigma), AlexaFluor 594 (Sigma), and AlexaFluor 546 (Invitrogen), as recommended by the manufacturer. For immunoblots, the secondary rat, mouse or rabbit antibodies used were coupled to alkaline phosphatase (Promega).

### Molecular cloning

To verify the predicted cDNA of DegP in RHΔ*Ku80* (type I) and in PruΔ*Ku80* (type II), total RNA was extracted from RHΔ*Ku80* or PruΔ*Ku80* tachyzoites using the NucleoSpin RNAII extraction kit (MACHEREY-NAGEL GmbH & Co.). Following RT-PCR using an oligo-dT and the Superscript first strand synthesis kit (Invitrogen), the specific full-length cDNA of *DegP* gene was amplified using primers ML220 and ML221 using the Phusion HF DNA polymerase (New England Biolabs). PCR amplification at the predicted size (2868bp) was cloned in the pCR-Blunt II-TOPO vector (Invitrogen) to generate TOPO-DegP^I^ (type I) and TOPO-DegP^II^ (type II). The ML220 and ML221 primers were designed to add the restriction sites *Bam*HI and *Mfe*I at the 5’ end and *Nsi*I and *Not*I at the 3’ end upon PCR amplification.

The pGEX-DegP-GST plasmid was designed to produce a recombinant GST-tagged DegP protein. The TOPO-DegP^I^ plasmid was digested by *BamH*I and *Not*I and the full-length cDNA sequence of *TgDegP* gene (2868bp) was cloned into the *BamH*I and *Not*I restriction sites of pGEX-4T-3 vector (GE healthcare).

The pET24a-DegP-His plasmidwas designed to produce a recombinant His-tagged DegP protein. An internal sequence of *DegP* cDNA was amplified using primers ML247 and ML248, digested by *Nde*1 and *Xho*1 and cloned into the *Nde*1 and *Xho*1 restriction sites of pET24a vector (Novagen).

The p*degP*-DegP-Ty plasmid was designed to express under the control of *degp* promotor a second copy of DegP^I^ fused with a Ty tag at the C-terminal. It was based on plasmid p*tub*-DegP-Ty, in which the tubulin promotor was replaced by the endogenous promotor of *degP*. The p*tub*-DegP-Ty plasmid was first designed to overexpress a second copy of DegP with a Ty tag at the C-terminal. To do so, the TOPO-DegP^I^ plasmid was digested by *Mfe*I and *Nsi*I and the full-length cDNA sequence of *TgDegP* gene (2868bp) was cloned into the *EcoR*I and *Nsi*I restriction sites of the pTUB8mycGFPPftailTY vector (a gift from Dominique Soldati-Favre), where it replaces the mycGFPPftail. p*DegP*-DegP-Ty was constructed by PCR amplification of p*tub*-DegP-Ty with primers ML234 and ML235 to create the deletion of the tubulin promotor as well as of the 108 first nucleotides encoding for the N-terminal 37 amino acids of DegP. The ML234 and ML235 primers were designed to add the restriction sites *Kpn*I and *Avr*II at the 5’and 3’ ends respectively upon PCR amplification. The template plasmids were eliminated by digestion with *Dpn*I, and a fragment corresponding to 1 kb of the 5’ non-coding region of DegP plus the 108 first nucleotides of the coding sequence of *DegP* was PCR amplified using primers ML232 and ML233, and then ligated with the PCR product obtained using ML234 and ML235 primers. This added an *Avr*II site six residues after the signal sequence of DegP.

The pDegP-V5 plasmid was designed to transfect mammalian cells with the *DegP* cDNA without the sequence signal. It was constructed by PCR amplification of the *DegP* cDNA from TOPO-DegP^I^ using primers ML277 and ML278, and cloning into *KpnI* and *XbaI* sites of pTRACER-A (Invitrogen).

The KO-DegP-HXGPRT plasmid was used to disrupt the *DegP* endogenous locus in both RHΔ*Ku80* and PruΔ*Ku80* strains. The primers ML981 and ML2048 were used to PCR amplify a 627bp fragment corresponding to the 5’ of the RHΔ*Ku80 DegP* gene containing the ATG, the first exon and part of the first intronic region (88 nucleotides). The PCR product was sub-cloned into the pCR-Blunt II-TOPO vector (Invitrogen), sequenced and then cloned KpnI/NsiI in the 3TY-HXGPRT vector [24]. The plasmid was linearized by *Pst*I prior to transfection. Correct integration of the disrupting plasmid was controlled by PCR using primers ML871/ML673. ML673 hybridizes to the TY tag present in the vector while ML871 is located 48bp upstream the *DegP* sequence cloned in KO-*DegP*-HXGPRT.

To generate KO-*DegP*^*II*^ revertant parasites, the chiRNA targeting the sequence GCTTCAGCATTGAAGACGTC from the HXGPRT cassette was cloned using the primers ML2248 and ML2249 in the pU6-universal plasmid [25].

All the plasmids were sequenced prior to transfection.

### Fluorescence staining of cells

For IFAs of transfected BHK-21 cells or intracellular parasites grown in host cells (HFF), cells were fixed with 4% formaldehyde in PBS and permeabilized with 0.1% Triton X-100 in PBS/3% BSA. Coverslips were blocked in PBS 10% FCS and proceeded further for IFA as previously described [26]. Samples were observed with a Zeiss Axioimager epifluorescence microscope equipped with an apotome and a Zeiss Axiocam MRmCCD camera driven by the Axiovision software (Zeiss), at the Montpellier RIO imaging facility. Images were collected and processed using Zeiss Zen software.

### Production of a recombinant DegP protein fused to GST and of a specific serum

*Escherichia coli* C41RIG competent cells were transformed with pGEX-DegP-GST or pGEX-DegP*-GST. The expression of DegP-GST or DegP*-His recombinant proteins were induced by adding 0.2mM IPTG at 37°C for 4h. Cells from 500mL of culture were harvested at 4000g for 20 min at 4°C and the pellet was resuspended in 25mL of PBS. The bacteria were lysed by French press (2 times with 2 tonnes / cm^2^) and the total lysate centrifuged at 9000 r.p.m during 20 minutes at 4°C. The *Tg*DegP-GST recombinant protein was abundantly recovered in the insoluble fraction. The DegP-GST protein was thus separated by SDS-PAGE and electro-eluted from the gel and used to raise polyclonal antibodies in rat as previously described [23].

### Immunoblots

Freshly released tachyzoites were harvested, washed in PBS and resuspended directly in SDS sample buffer. All gels were run under non-reducing conditions except otherwise stated in the figures legends. 5.10^6^ parasites were loaded in each lane. Transfer and immunodetection were done as previously described [19]. DegP*-His recombinant protein was purified using a nickel-agarose column in native or denaturing condition and according to the QIAGEN protein purification handbook.

### Plaque and intracellular growth assays

Plaque and intracellular growth assays were performed as previously described [19]. To assess intracellular growth, only the vacuoles containing 2 parasites or more were scored.

### Two-color invasion assays

Two-color invasion assays were performed as previously described [27]. Briefly, 5.10^6^ freshly released tachyzoites from the control strain (RHΔ*Ku80* or PruΔ*Ku80*) or from the mutant strains (KO-*DegP*^*I*^ or KO-*DegP*^*II*^) were added to HFF grown on glass coverslips, synchronized on ice during 20 min and subsequently allowed to invade for 5 min in invasion buffer. Invasion was stopped by fixation in 4% PAF in HBSS and parasites were further processed for IFA. A first immunodetection with the mouse mAb T4 1E5 anti-SAG1 in 2% FCS/HBSS was performed to detect extracellular parasites. After permeabilization with 0.1% saponin for 15 min, a second IFA was performed using rabbit anti-ROP1 antibodies to label the parasitophorous vacuole of intracellular parasites. Extracellular and intracellular parasites were counted on 40–50 fields per coverslip from three coverslips.

### *In vivo* experiments

To assess the KO*DegP*^*I*^ virulence *in vivo*, 100 tachyzoites from the KO-*DegP*^*I*^ or RHΔ*Ku80* were intraperitoneally injected (i.p) into 20 females 8 weeks-old Swiss mice. To assess the KO-*DegP*^*II*^ virulence *in vivo*, 12–16 week-old Swiss mice (Charles River, France) were infected by i.p. injection of 10^5^ or 1 million tachyzoites freshly harvested from cell culture. To test the virulence of PruΔ*Ku80* or KO-*DegP*^*II*^ in NOD/Shi-scid/IL-2Rγ^null^ (NOG) Severe Combined Immunodeficiency), 12 weeks mice were infected with 10^5^ parasites freshly harvested from cell culture. Invasiveness of the parasites was evaluated by simultaneous plaque assay of a similar dose of parasites on HFFs. Mouse survival was monitored daily until their death, end-point of all experiments. The immune response of surviving animals (except for NOG) was tested following eye pricks performed on day 7 post infection. Sera were tested by Western blotting tachyzoite lysates. Data were represented as Kaplan and Meier plots using GraphPad Prism version 4.00 for Windows (GraphPad Software).

### Bioinformatic domain prediction

SignalP software (http://www.cbs.dtu.dk/services/SignalP/) was used to predict signal peptide. The catalytic domain was predicted by using Pfam software (http://pfam.xfam.org/) and the PDZ domains were mapped using supfam software (http://supfam.org/SUPERFAMILY/). Alignment was performed using ClustalW2 (http://www.ebi.ac.uk/Tools/msa/clustalw2/).

### Statistical analysis

All results are presented as mean values with standard deviations shown as error bars. Two-tailed unpaired Student’s t tests (for plaque assays, invasion and replication assays) were used appropriately to determine statistical significance using GraphPad software (version 7) and a P value 0.05 was considered significant. For *in vivo* experiments, levels of significance were determined with the Logrank test using GraphPad.

### Oligonucleotides used in this study

ML220: 5’-GGATCCCAATTGATGTTGCTCCTTCTGTCACT-3’
ML221: 5’-GCGGCCGCATGCATTGAGGAAGTAAGAGCGGTCTCTCA-3’
ML232: 5’-GGGGTACCCCGAAGACCACAGCCCCAGA-3’
ML233: 5’-ATGCATCCTAGGTAACAGAGACGAAGCCTCCG-3’
ML234: 5’-GGGGTACCCAATTCGCCCTATAGTGAAGTC-3’
ML235: 5’-ATGCATCCTAGGACTCCTGAACCTTCTGAGACC-3’
ML247: 5’-CATATGCAGAGTGTGTGCAGGCTCAAGA-3’
ML248: 5’-CTCGAGCAACAGGAGTTCTTCTTCCGA-3’
ML277: 5’-GGGGTACCATGGAGCAGAAGCTCATCTCCGAGGAGGAC-3’
ML278: 5’- GCGGTACCGAGGAAGTAAGAGCGGTCTC-3’
ML673: 5’-ATCGAGCGGGTCCTGGTTCGTGTGGACCTC-3’
ML871: 5’-TCTAGAATGGCGGCGTTCCACCGG-3’
ML981: 5’-GGTACCATGTTGCTCCTTCTGTCACTG-3’
ML1022: 5’-CTCGGGTTGTTTCACCAACGACG-3’
ML2048: 5’-ATGCATGTCTCTACTTTACTAACTTCAC-3’
ML2248: 5’-AAGTTGCTTCAGCATTGAAGACGTCG-3’
ML2249: 5’- AAAACGACGTCTTCAATGCTGAAGCA-3’

## Results

### *Tg*DegP is a rhoptry protein

**Fig 1:**
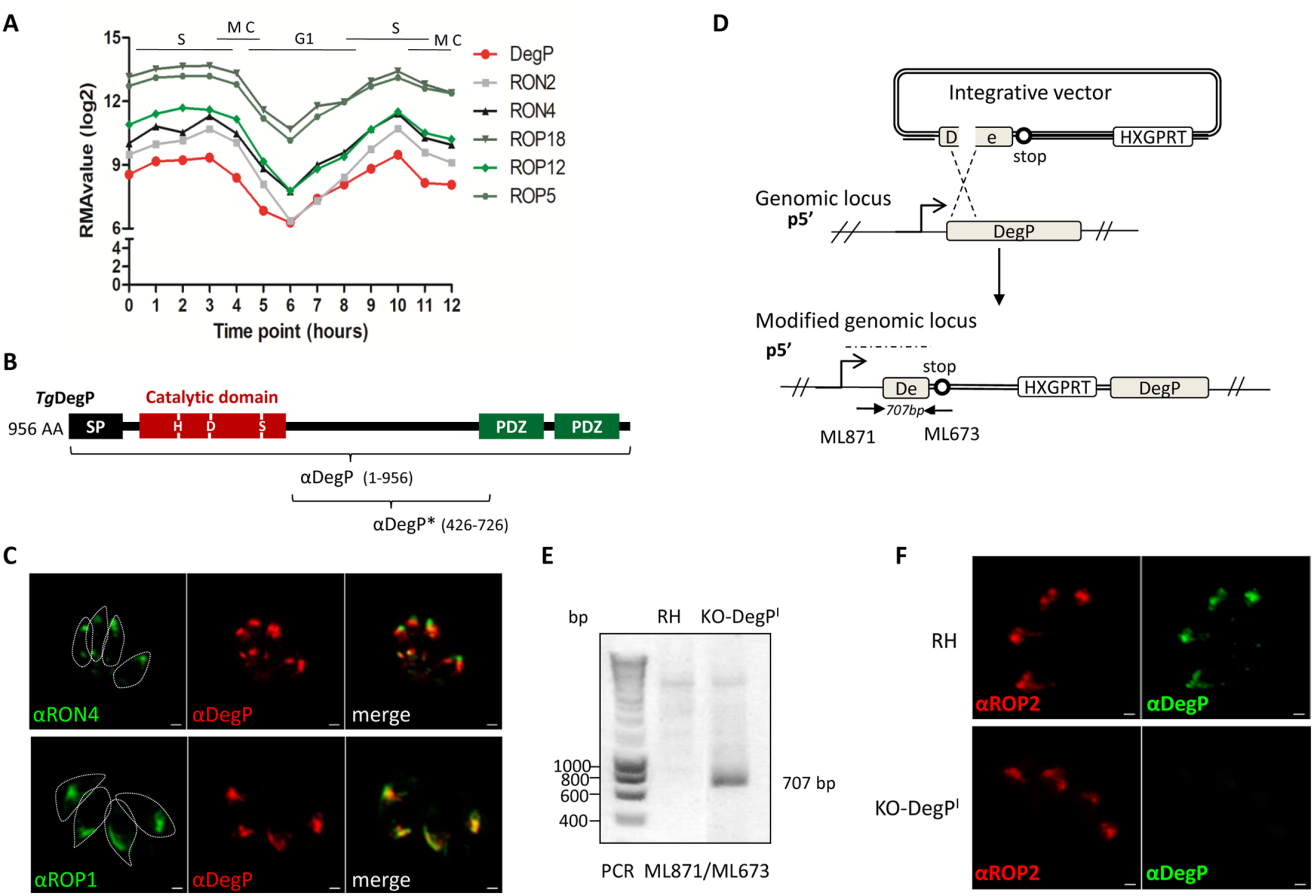
*Tg*DegP is a rhoptry bulb protein. (A) Graph representing microarray data of transcripts encoding some known rhoptry proteins (RON2, RON4, ROP5, ROP12, ROP18) along with DegP transcript hourly following thymidine synchronization (from [29]). (B) Primary structure of *Tg*DegP. *Tg*DegP is a putative serine protease of 956 amino acids belonging to HtrA family. Signal peptide is shaded in black (SP), the catalytic domain is represented in red and H, D and S indicate the positions of the catalytic triad. The two PDZ domains are represented in green. The rat anti-DegP has been made against the complete DegP recombinant protein and the mouse anti-DegP* has been generated against a recombinant protein encompassing residues 426 to 726. (C) Immunofluorescence performed on intracellular parasites using the anti-DegP serum, anti-RON4 and anti-ROP1. On parasites fixed with methanol (upper panel), the rabbit polyclonal anti-RON4 antibodies revealed the neck of the rhoptry. On paraformaldehyde-fixed parasites (lower panel), the DegP protein co-localizes with the bulbous ROP1 protein (labeled with the rabbit polyclonal anti-ROP1) in the bulb of the rhoptry. Parasite boundaries are represented by dashed lines. Scale bar = 1μm. (D) Generation of a knock-out *DegP* parasites in type I strain RHΔ*Ku80*. Scheme depicting the strategy used to obtain a KO-*DegP*^*I*^ cell line. The promoter of *TgDegP* is represented by an arrow. Integration of the plasmid by a single homologous recombination at the *DegP* locus results in a truncated version of DegP driven by the endogenous *DegP* promoter while the *DegP* coding sequence is now promoter less. HXGPRT: hypoxanthine guanine phosphoribosyl transferase selection marker. The solid arrows represent the primers used to verify the integration of the integrative vector and the expected size of the fragment is shown in italics. ML673 hybridized to a sequence specific of the vector, while ML871 is located in the 5’UTR of *DegP* outside the cloned fragment. (E) PCR verification of the correct integration of the vector at the endogenous *DegP* locus. The primers ML673 and ML871 are used in this PCR. The recombined locus was detectable only in transgenic parasites KO-*DegP*^*I*^, as shown by a specific amplification of a 707 bp fragment that is not amplified in the parental strain. (F) Immunofluorescence performed on KO-*DegP*^*I*^ or RHΔ*Ku80* cell lines with the rat anti-*Tg*DegP antibodies and rabbit anti-ROP2 antibodies as control for rhoptry staining. The DegP labelling is absent in the KO-*DegP*^*I*^ transgenic parasites. ROP2 staining remains unchanged in the mutant. Scale bar = 1 μm.

**Fig 2:**
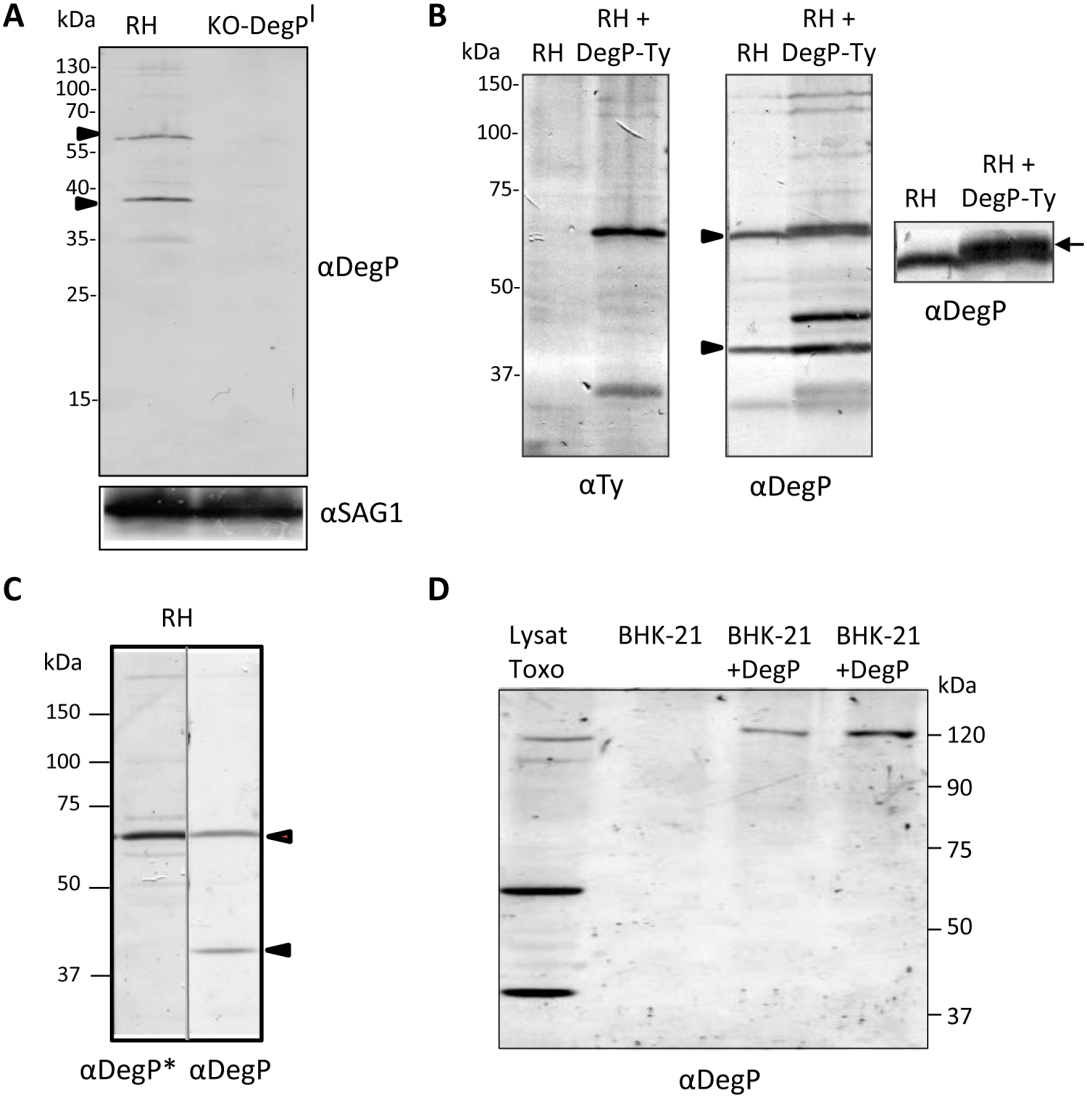
DegP is subjected to proteolytic maturation. (A) Western blot analysis of RHΔ*Ku80* lysate with anti-DegP serum reveals a complex profile. The specificity of the bands was assessed using KO-*DegP*^*I*^ parasite lysate. Two major bands are observed around 60 kDa and 40 kDa respectively and are indicated by black arrows. Molecular weights are indicated. Anti-SAG1 antibody was used as loading control. (B) Western blots analysis of RH strain expressing an ecto-copy of DegP fused to a Ty tag at the C-terminal end. Right panel: anti-DegP antibodies. Left panel: anti-Ty antibodies. An additional band around 60 kDa with a slight shift is visible by staining the RH strain expressing DegP-Ty with anti-DegP antibodies (see arrow on enlarged panel on the right). (C) Western blot profiles of DegP under reduced conditions using anti-DegP* (426–726) serum. Anti-DegP* reveals only the 60kDa band. (D) Exogenous expression of *Tg*DegP in mammalian cells. Western blot analysis of BHK-21 cells transfected with the cDNA of DegP under the control of promotor EF-1α. Lane 1 correspond to *T*. *gondii* lysate; lane 2 was control BHK-21 cells transfected with empty plasmid pTRACER-A, lane 3 was lysate of BHK-21 cells transfected with 1μg of plasmid p*DegP*-V5 and lane 4 was lysate of BHK-21 cells transfected with 2 μg of plasmid p*DegP*-V5. The profile of *Tg*DegP expressed in mammalian cells is restricted to a unique form corresponding to the full-length protein.

A previous proteomic analysis of a highly purified fraction of rhoptries from *Toxoplasma* revealed peptides corresponding to a protease (TGME49_262920), that is close to chymotrypsin [28]. However, its presence in the rhoptry compartment has not been yet validated experimentally. TGME49_262920 encodes a 956 amino acid protein, which displays a conserved protease domain in the N-terminal part of the protein, with the critical catalytic triad (aspartic acid, histidine, and serine residues) typical for serine proteases (Fig 1A). In addition, two PDZ domains are present in the C-terminal end of the protein. According to this modular architecture combining a protease domain with one or more C-terminal PDZ domains characteristic of Deg-like serine proteases, this protein was named *Tg*DegP [15]. *Tg*DegP harbors a predicted signal peptidase cleavage site after Ala30, indicative of trafficking along the secretory pathway. Moreover, its cell cycle time course transcription (ToxoDB.org) shows a maximum in S to M phase and then dramatically decreases in early G1 before picking again in the next S phase; a mRNA periodicity typical of rhoptry proteins [29] (Fig 1A). To validate *Tg*DegP as a rhoptry protein, we experimentally assessed the location of *Tg*DegP in RHΔ*Ku80* type I strain by producing a specific serum (named anti-DegP) directed against the full length of *Tg*DegP recombinant protein fused to GST and produced in *Escherichia coli* (Fig 1B). By IFA, the anti-DegP serum recognizes an antigen co-localizing with the rhoptry bulbous protein ROP1 but distinct from the rhoptry neck marker RON4 in intracellular parasites (Fig 1C). The specificity of the serum was confirmed using a *TgDegP* knock-out parasite line, named KO-*DegP*^*I*^ (I, for KO in type I strain) (Fig 2A). KO-*DegP*^*I*^ was engineered using a disruption strategy by single homologous recombination as previously described [21](Fig 1D). Following vector integration, a truncated version of DegP that does not contain the full catalytic domain but containing only the first 178 residues might be expressed. The correct integration of the vector in the *DegP* locus was verified by PCR (Fig 1E) and the subsequent loss of DegP protein in KO-*DegP*^*I*^ parasites was verified by IFA (Fig 1F) and Western Blot analysis (Fig 2A).

### *Tg*DegP undergoes proteolytic maturation

*Tg*DegP has a predicted molecular mass of 104 kDa. Immuno-detection with the anti-DegP serum on a *T*. *gondii* tachyzoite lysate of the RHΔ*Ku80* strain under non-reduced (Fig 2A) and reduced conditions (Fig 2C) revealed two major products migrating around 60 kDa and 40 kDa; additional weaker bands with inconsistent intensities depending on immunoblots were also obtained. Western-blot on KO-*DegP*^*I*^ parasite lysate with anti-DegP antibodies showed the concomitant extinction of the 60 kDa and 40 kDa bands (Fig 2A), confirming the specificity of these products. Therefore, the observed profile suggests that *Tg*DegP undergoes post-translational maturation, a characteristic shared by most of *T*. *gondii* rhoptry proteins described so far [30]. We then expressed an ectocopy of DegP fused in C-terminal with a sequence encoding a Ty epitope. A major band at 60 kDa and a faint band around 120 kDa were detected with anti-Ty antibody (Fig 2B), indicating that the 60 kDa fragment corresponds to the C-terminal part of DegP whereas the 120 kDa corresponds likely to the full length protein. Interestingly, in cells overexpressing DegP-Ty, the intensity of the additional bands increases compare to the parental strain (like the one observed around 45kDa) (Fig 2B) that might be attributed to a decrease in stability of DegP following tag addition. We further produced a serum against a recombinant protein encompassing residues 426 to 726 (anti-DegP*) (Fig 1A), which does not include the catalytic domain of DegP (corresponding to residues 147 to 379). The anti-DegP* labeled the 60 kDa band but failed to detect the 40 kDa (Fig 2C). This result is consistent with the results obtained with the Ty-tagged version of DegP and confirms that the 60 kDa corresponds to the C-terminal part of the protein whereas the 40 kDa band likely corresponds to its N-terminal region, encompassing the catalytic domain.

Interestingly, when the full length cDNA of DegP is expressed in mammalian cells, a single band migrating above 120 kDa is observed (Fig 2D). The absence of DegP processing in mammalian cells indicates that the post-translational maturation depends on its expression in *Toxoplasma*.

### KO–DegP parasites in type I RH strain exhibit normal growth and virulence in mice

**Fig 3:**
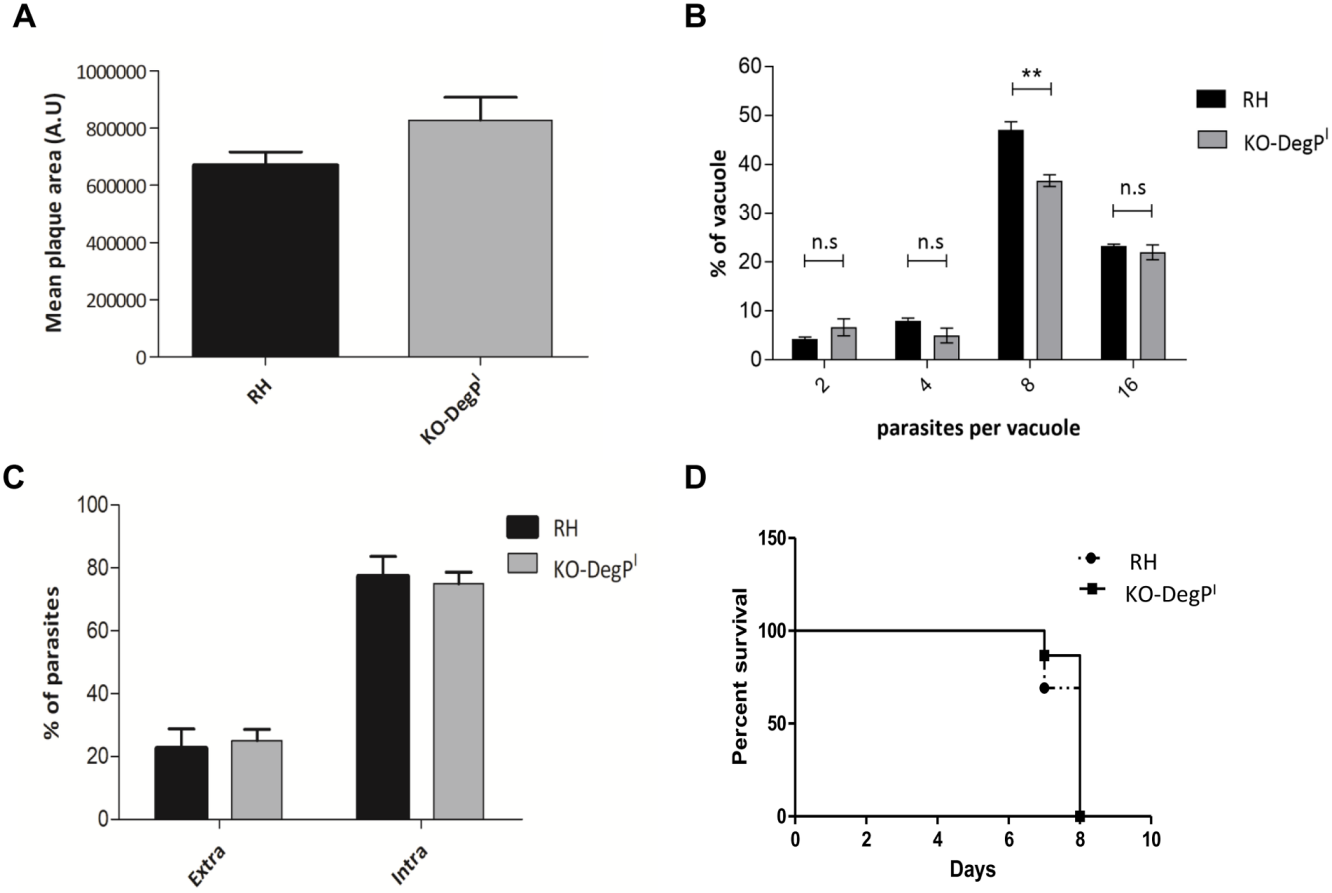
*Tg*DegP is dispensable for *in vitro* and *in vivo* survival of the type I strain. (A) Size of the plaques resulting from the lysis of host cells infected with RHΔ*Ku80* or KO-*DegP*^*I*^ parasites and grown for seven days. A.U.: arbitrary units. Values represent means ± SEM, n = 3, from a representative experiment out of 3 independent assays (p = 0.095; unpaired t-test). (B) Intracellular growth rate was assessed by counting the numbers of parasites per vacuole after 20 h infection of HFF cells with RHΔ*Ku80* or KO-*DegP*^I^ parasites. Data Values represent means ± SEM, n = 3, from a representative experiment out of 3 independent assays (0.001<**<0.005; unpaired t-test). (C) Host cell invasion efficiencies of RHΔ*Ku80* or KO-*DegP*^*I*^ strains determined by a two-color staining protocol that distinguishes intracellular from extracellular parasites. Data are mean values ± SEM determined by triplicate assays, performed in three separate experiments (p = 0.75; unpaired t-test). (D) Mouse survival after i.p injection of 100 tachyzoites of RHΔ*Ku80* or KO-*DegP*^I^ parasites was monitored daily for 10 days. N = 13 mice for RHΔ*Ku80* and N = 15 mice for KO-*DegP*^*I*^ strains. Representative data out of 3 experiments. KO-*DegP*^*I*^ strain is not statistically less virulent than RHΔ*Ku80* (p = 0.27, by Logrank test).

Isolation of stable parasite clone that does not express functional DegP (Fig 1) demonstrates that the protein is not strictly essential for the parasite *in vitro*. The dispensability of DegP was further confirmed by plaque assay in which KO-DegP^I^ parasites displayed no significantly smaller plaques than the parental RHΔ*Ku80* (Fig 3A). In order to highlight potential more subtle defects, we then assessed the ability of the KO-DegP^I^ parasites to invade and replicate in HFF cells. Results presented in Fig 3B and 3C show no significant difference in the replication rate and invasion between the parental and the mutant cell lines. Considering the role of some rhoptry bulb proteins in the modulation of host immune responses, we then assessed the effect of DegP depletion on *in vivo* virulence in a murine model. After i.p injection of 100 tachyzoites into BALB/c mice, we observed a similar survival pattern between mice infected with the KO-*DegP*^*I*^ parasites or those infected with the parental RHΔ*Ku80* strain.

Overall, our data show that the loss of *Tg*DegP does not impede the intracellular development of the type I RH strain in HFF cells or its virulence in a mouse model.

### Deletion of DegP in type II Prugniaud strain dramatically affects the parasite virulence in mice

**Fig 4:**
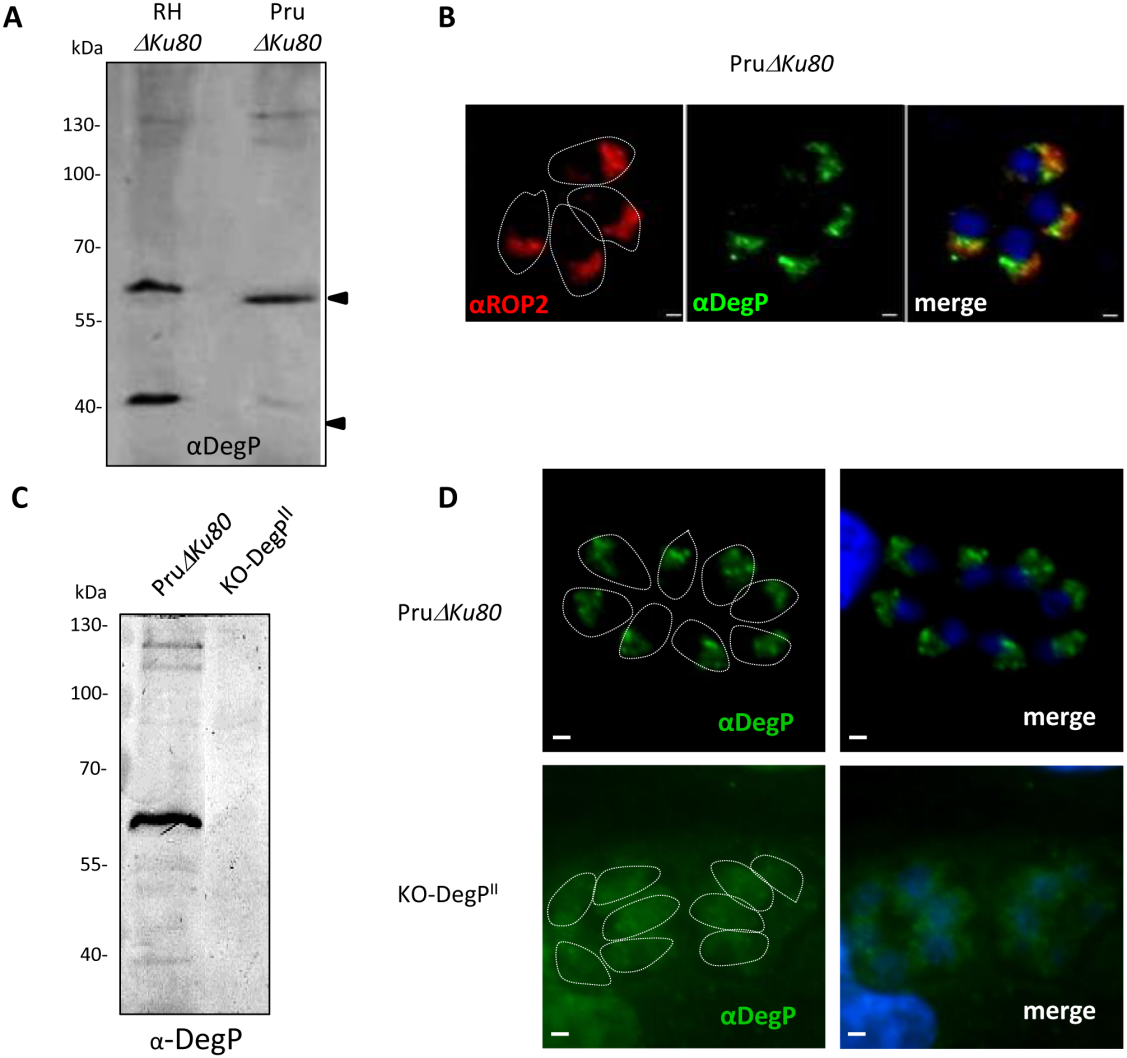
Expression of *Tg*DegP in the type II PruΔ*Ku80* strain. (A) Western blot detection of *Tg*DegP in the Prugnaud strain (Pru), a representative member of the Type II *Toxoplasma gondii* genotype. Blot was revealed using the polyclonal anti-DegP antibody. Molecular weights are indicated. Note that the intensity of the 40kDa band is faint the PruΔ*Ku80* strain. (B) Immunofluorescence performed on PruΔ*Ku80* intracellular parasites using the anti-DegP and anti-ROP2 antibodies. As observed in the type I strain, *Tg*DegP^II^ colocalizes with ROP2 in the bulb of the rhoptry. Parasite boundaries are represented by dashed lines. Scale bar = 1μm. Western blot (C) and immunofluorescence (D) performed on PruΔ*Ku80* and KO-*DegP*^II^ cell lines with the rat anti-*Tg*DegP. The DegP labelling is absent in the KO-DegP^II^ transgenic parasites. Parasite boundaries are represented by dashed lines. Scale bar = 1 μm.

As rhoptry proteins are pivotal players in the strategies developed by type I and II strains to establish an infection, we further investigate the role of DegP in a type II strain (Pru). DegP is conserved in type II strain. The gene shows low level of polymorphisms between strains with a strong conservation in the sequences encoding the predicted functional domains (protease and PDZ) while few SNPs are found both in the 5’end of the gene and in the fragment between the sequences coding for protease and PDZ domains. We show that DegP^II^ is well expressed in type II PruΔ*Ku80* strain (Fig 4A) and harbors the same localization as in type I strain i.e in the bulb of the rhoptries (Fig 4B). Western blot analysis showed that TgDegP^II^ undergoes a similar maturation profile than in the type I RH strain with the notable difference that the intensity of signal for the 40 kDa product was consistently lower in PruΔ*Ku80* compareto RHΔ*Ku80* strain (Fig 4A).

**Fig 5:**
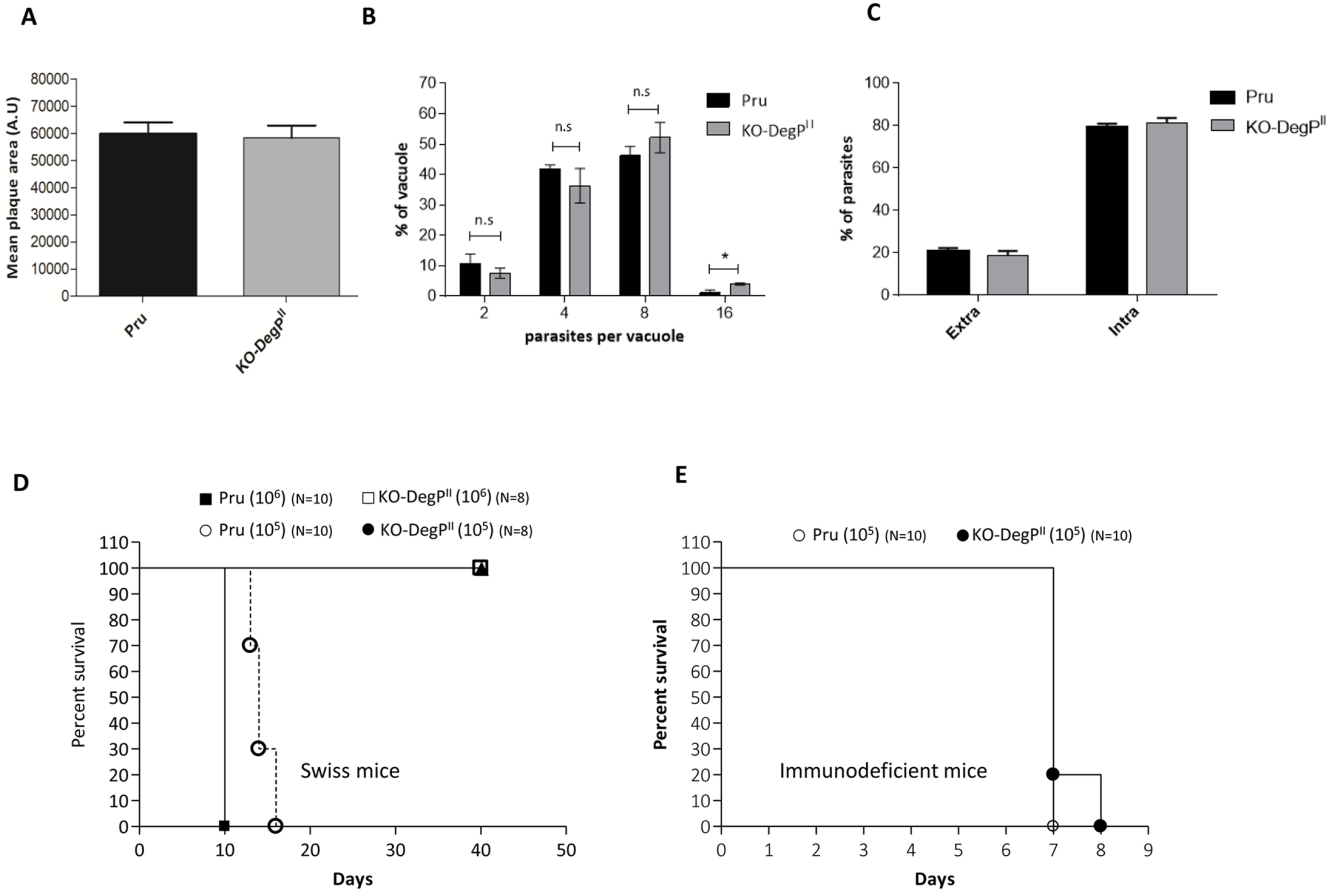
Deletion of DegP in type II strain affects the *in vivo* virulence of the parasite. (A) Confluent monolayers of human fibroblasts were infected with PruΔ*Ku80* or KO-*DegP*^*II*^ parasites and grown for seven days. Quantification of the size of the lysis plaques is shown in the graph. The size of the lysis plaques is similar between control (PruΔ*Ku80*) and KO-*DegP*^*II*^ strains. A.U.: arbitrary units. Values represent means ± SEM, n = 3, from a representative experiment out of two independent assays (p = 0.82; unpaired t-test). (B) Intracellular growth rate was assessed by counting the numbers of parasites per vacuole after 20 hours infection of HFF cells with PruΔ*Ku80* or KO-*DegP*^*II*^ parasites. Data Values represent means ± SEM, n = 3, from a representative experiment out of two independent assays (0.05<*<0.1; unpaired t-test). (C) Host cell invasion efficiencies of RHΔ*Ku80* or KO-*DegP*^*II*^ strains determined by a two-color staining protocol that distinguishes intracellular from extracellular parasites. Data are mean values ± SEM determined by triplicate assays, performed in two separate experiments (p = 0.48; unpaired t-test). (D) Mouse survival was monitored daily for 40 days after i.p injection of 10^5^ or 10^6^ parasites of PruΔ*Ku80* or KO-*DegP*^*II*^ parasites. N = 10 mice per group. Representative data out of 2 experiments. The immune response of surviving animals was tested by Western blotting against PruΔ*Ku80* tachyzoite lysates. In Swiss mice, KO-*DegP*^*II*^ strain is statistically less virulent than RHΔ*Ku80* (p<0.001, by Logrank test for10^5^ and 10^6^ i.p injections). (E) Mouse survival in immunodeficient NOG mice after i.p. injection of 10^5^ parasites PruΔ*Ku80* or KO-*DegP*^*II*^ parasites. N = 10 mice per group. Representative data out of 2 experiments. In NOG mice, the virulence of KO-*DegP*^*II*^ and RHΔ*Ku80* was not statistically different (p = 0.146, by Logrank test).

To further expand our comparative approach in both types of strains, we generated a KO-*Deg*P in the PruΔ*Ku80* strain (KO-*DegP*^*I*I^) (Fig 4C and 4D; S1A and S1B Fig) using the same genetic disruption approach as in type I strain. The disruption of the *DegP* gene in the type II strain does not affect the size of the lysis plaques in HFF cells, or the replication rate and the ability of the parasites to invade HFF cells (Fig 5A–5C). However, a clear impact of DegP^II^ removal was observed on the virulence *in vivo* (Fig 5D). All the Swiss mice survived i.p infection with 10^5^ tachyzoites of the KO-*DegP*^*I*I^ strain for at least 40 days, while mice infected with the wild-type PruΔ*Ku80* strain died within around 15 days. Inoculation of mice with a higher dose of parasites (10^6^) confirmed the defect in virulence of KO*DegP*^II^ parasites (Fig 5D).

**Fig 6:**
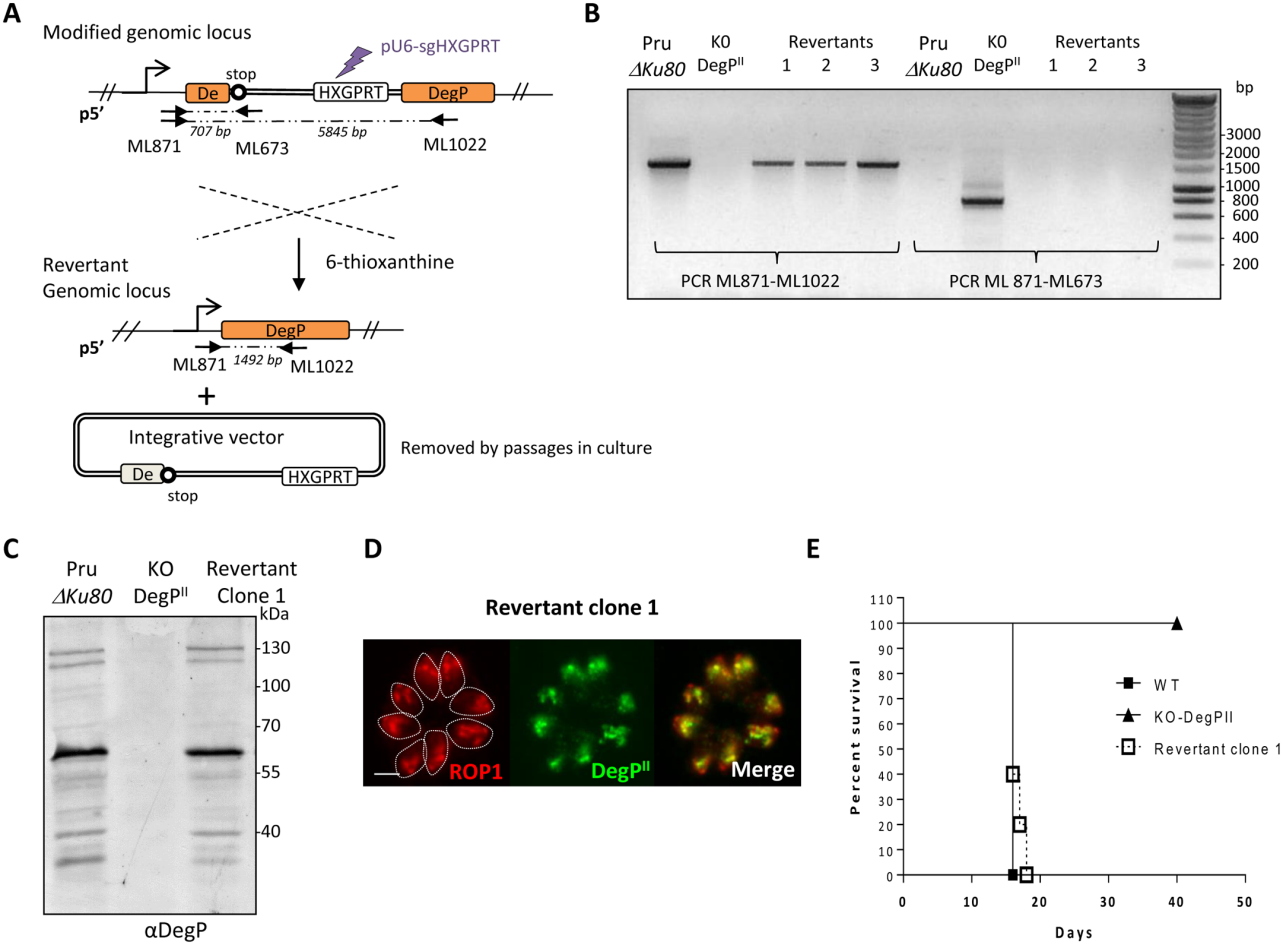
The repair of the *DegP* locus in KO-*DegP*^*II*^ restores virulence in mice. (A) Scheme depicting the strategy used to select parasites that have removed by single recombination the knock-out plasmid in KO-*DegP*^*I*^ cell line. The purple arrow indicates the site targeted to induce a double strand break in *HXGPRT* locus using plasmid pU6-sgHXGPRT. In absence of donor fragment containing *HXGPRT* homology regions for double strand break repair and culture in presence of 6-thioxanthine (selection for loss of *HXGPRT* gene), this strategy fosters the selection of single recombination event at the DegP homology regions (in orange), and reconstitution of wild-type *DegP* locus. The promoter of *TgDegP* is represented by an arrow. Primers ML871 and ML673 are used to check the interrupted *DegP* locus. Primers ML871 and ML1022 are used to control reversion of the mutation. ML673 hybridized to a sequence specific of the vector, ML871 is located in the 5’UTR of *DegP* (outside the cloned fragment in the integrative plasmid) and ML1022 hybridized in exon 2 of *Deg*P (outside the cloned fragment in the integrative plasmid). The solid arrows and the expected sizes of the fragments are shown in italics. (B) PCR verification of the correct *DegP* locus in revertant. The recombined locus (specific amplification of a 707 bp fragment with the primers ML673 and ML871) was detectable only in transgenic parasites KO-*DegP*^*II*^, as shown by amplification of a 707 bp fragment with primers ML871 and ML673. A fragment of 1492bp corresponding to the wild-type genomic locus is amplified in the parental strain PruΔ*Ku80* and in the selected revertant clone, but is absent in KO-*DegP*^*I*I^ strain. (C) Western blot and (D) Immunofluorescence performed on PruΔ*Ku80*, KO-*DegP*^*II*^ and revertant cell lines with the rat anti-*Tg*DegP antibodies and rabbit anti-ROP1 antibodies confirm the DegP labelling in revertant clone. Parasite boundaries are represented by dashed lines. Scale bar = 5 μm. (E) Mouse survival after i.p injection of 10^5^ parasites of PruΔ*Ku80*, KO-*DegP*^*II*^ and revertant parasites was monitored daily for 40 days. N = 9 mice for PruΔ*Ku80*, N = 11 mice for KO-*DegP*^*II*^ and N = 10 for revertant strains. Representative data out of 2 experiments. The revertant parasites kill mice similar to wild-type PruΔ*Ku80* strain.

To ascertain that the observed *in vivo* phenotype is solely due to the disruption of the *DegP* locus, we tried to complement the KO-DegP^II^ strain with an additional copy of the coding sequence. As all attempts to genetically complement the mutant, using different strategies (random integration into the genome, targeting the UPRT locus) and different selection markers failed, we opted for an alternative strategy. As previously mentioned, disruption of the *DegP* locus was obtained by single recombination and insertion of a linear plasmid containing the HXGPRT gene (Fig 1C). Thus, another strategy was adopted consisting in the generation of revertant parasites that have lost the integrative plasmid (i.e reversion) and restored the *DegP* locus. Expression of HXGPRT in parasites confers resistance to mycophenolic acid (MPA) but becomes lethal for the parasite in presence of 6-thioxanthine (Fig 6A), allowing to select revertant in presence of 6-thioxanthine. To increase the efficiency of removing the plasmid and HXGPRT cassette, parasites were transfected with a pU6-Universal plasmid [25] containing the Cas9-YFP and a 20 bp protospacer sequence targeting the coding sequence of *HXGPRT* (Fig 6A). Revertant clones were selected in presence of 300 μg/ml of 6-thioxanthine and their sensitivity to MPA-xanthine was confirmed. The restoration of the *DegP* locus was verified by PCR (Fig 6B) and the expression of DegP was confirmed by immunofluorescence (Fig 6D) and western blot (Fig 6C) using anti-DegP antibodies. In mouse model, we then successfully showed that the *in vivo* virulence of revertant was fully restored (Fig 6E).

All together these results clearly pointed on a specific role of DegP in virulence of the Prugniaud type II strain.

### Infection of immuno-deficient mice suggests a role for DegP^II^ in immune evasion

During infection, *Toxoplasma* is known to modulate numerous host cell processes, including lipid metabolism, protein synthesis, cell signaling, amino acid metabolism, apoptosis as well as the immune response by secreting parasite effectors [31]. To further discriminate whether DegP^II^ might play a role in the subversion of host cell protecting immune processes, we used immunodeficient mice NOG. These mice are homozygous for the *SCID* mutation and were generated by targeted disruption of the interleukin *(IL)-2Rγ* gene. Since the γ-chain is common to the receptors for IL-2, IL-4, IL-7, IL-9, IL-15, and IL-21, NOG mice lack B- and T-cell development, have impaired macrophage and NK-cell functions and have a severe reduction in interferon-γ production from dendritic cells [32]. Following infection of NOG mice with 10^5^ parasites, we observed the same rate of mortality of mice whether they were infected with the wild-type or KO-*DegP*^*II*^ parasites (Fig 5E). In conclusion, these results showed that, in the absence of an efficient immune response setting, KO-*DegP*^*II*^ parasites show the same ability to cause lethal infection than WT parasites suggesting that DegP^II^ is important for virulence in a fully immune competent environment.

## Discussion

To date, two distinct type of proteases have been discovered in the rhoptry bulb: a subtilisin-like serine protease (*Tg*SUB2) [33] and an insulinase-like protein named Toxolysin-1 (TLN1) [34] that belongs to the M16 metalloprotease family. As most of the subtilisins, *Tg*SUB2 is autocatalytically processed [33] and has been proposed to be the maturase of rhoptry proteins [33,35], while the function of TLN1 is unknown. A third protease belonging to Deg-like serine proteases had been found in the rhoptry proteome [28], but its formal association with the organelle and its precise sub-compartment localization were missing. Here, we show that *Tg*DegP is a rhoptry bulb protein and perform a comparative functional analysis by generating mutant harboring a truncated version of DegP (lacking the protease and PDZ domains) in both type I and type II strains. We notice any defect in lytic cycle in absence of the protease in both strains, indicating that DegP does not play any role for invasion, replication and egress *in vitro* as predicted by a genome-wide CRISPR screen in which DegP deletion was found neutral for *in vitro* growth in fibroblasts [36]. In contrast, a marked defect in virulence for KO-DegP parasites in a type II strain was observed in immune-competent mice where KO-*DegP*^*I*I^ parasites failed to establish a lethal infection *in vivo*. Whereas we were unable to detect the truncated version of DegP by western blot in KO-*DegP*^*I*^ parasites (Fig 2A) suggesting that it is not stably expressed, it remains possible that a small proportion of truncated DegP participates to the observed *in vivo* phenotype. In the method adopted to generate the KO-DegP, the insertion of the integrative vector is susceptible to disturbs *DegP* neighboring genes. However, it seems unlikely to be the case as *DegP* proximal genes are reported to strongly impair *in vitro* growth of *T*. *gondii* in fibroblasts [36], a phenotype that we do not observed in both KO-*DegP* parasites. Interestingly, the lack of virulence of KO-DegP^II^ parasites was not observed in SCID mice where all immunocompromised mice were killed by KO-DegP^II^ at a similar rate as WT parasites. Rather than a defect in virulence due to nutrient limitation (as observed for uracil phosphoribosyltransferase mutant [37]), the ability of KO-*DegP*^*II*^ parasites to establish a lethal infection in a permissive immune environment (SCID mice) suggests a role for DegP^II^ in the complex mechanism of resistance to the immune response. How DegP exerts its functions remains to be determined and several scenarios might be envisaged. Similar to most of the rhoptry proteins studied so far, DegP might be secreted into the host cell during invasion and target a host cell pathway. We were unable to detect DegP post-secretion in the host cell either by immunofluorescence or by using a reporter system adapted for the detection of *Toxoplasma* secreted proteins [38], precluding to know the fate of DegP following invasion. The possible secretion of DegP into the host cells to act as an effector per se requires further investigations. We cannot exclude that DegP might also play an indirect role on rhoptry effectors. It could be important for processing, functions and trafficking of other rhoptry proteins or effectors. Since DegP proteins are reported to function both as molecular proteases and chaperones (see Discussion below), a third hypothesis could be that TgDegP functions as a chaperone specifically in the stress conditions encountered *in vivo* or in specific cell type. Supporting this hypothesis, *P*. *falciparum* homologous DegP, PfDegP, has been recently proposed to confer protection against thermal/oxidative stress [39]. PfDegP exists as a complex with parasite-encoded heat shock protein 70, iron superoxide dismutase and enolase and its expression is significantly induced in parasite culture upon heat shock/oxidative stress. More broadly, in intracellular bacteria such as *S*. typhimurium, *L*. *monocytogenes*, and *Y*. *enterocolitica*, *htrA* mutants show increase sensitivity to oxidative agents leading to reduced survival in macrophages. Hence, attenuated virulence of these pathogens will be attributed to a decreased ability to persist in the hostile environment of macrophages, which like for *Toxoplasm*a is fundamental to trigger successful infection [40,41].

Deg proteases are a highly conserved family of proteins from bacteria to human composed of a N-terminal protease domain and one or two C-terminal PDZ domains. They can function as both a molecular protease and a chaperone, without any requirement for ATP hydrolysis [42]. The switch from molecular chaperone to protease activity is regulated by temperature and also by substrate recognition [43,44]. While the protease domain is required for both protease and chaperone activities, the role of the PDZ domains is far less understood. To date, they are proposed to play a role in substrate recognition [45]. *Tg*DegP follows the same organization as Deg proteases with two PDZ domains. The PDZ and the protease domains are identical between type I and type II strains. However, we do observe changes at the protein level post-processing with the fragment corresponding to the catalytic domain (40 kDa fragment) underrepresented in the Prugniaud type II strain compared to the type I strain RHΔ*Ku80*. Unfortunately, despite multiple attempts we were unable to complement the strain either with a WT or a protease dead version of DegP precluding any conclusion regarding the importance of its protease function. As DegP/HtrA proteases harbors an allosteric activation mechanism in other organisms, it is possible that the stoichiometry between the PDZ and catalytic domains is an important parameter to regulate the activity of the protein with the possibility that the function of *Tg*DegP in type II strain might reside in the ‘noncatalytic’ PDZ domains.

Genome mining and multiple sequence alignment identified homologs of *Tg*DegP in most Apicomplexa, including *Eimeria*, *Neospora*, *Hammondia*, *Babesia*, *Theileria* and *Plasmodium spp*. with the notable exception of rodent malaria parasites [15]. Multiple copies of trypsin genes have been found in *Toxoplasm*a and *Plasmodium*. For instance, *Toxoplasma* contains four *DegP*-like genes (TGME49_262920 (this study), TGME49_290840, TGME49_277850, TGME49_318290) and *P*. *falciparum* has two (Pf_MAL8P1.12 (named *Pf*DegP [39]) and Pf_MAL8P1.98). Phylogenic analysis supports two different evolutionary origins for these trypsins. A group of apicomplexan trypsins clusters with plants, metazoan and cyanobacterial trypsins and contains *Tg*DegP (TGME49_262920), TGME49_290840 and *Pf*DegP (Pf_MAL8P1.12), the two copies in *Toxoplasma* arising probably by gene duplication [15]. Sequence alignment of Apicomplexa trypsins reveals the presence of a large insertion in the central region of *Tg*DegP, between the protease and PDZ domains (S2A Fig). This insertion is only present in Coccidian parasites, and moreover is divergent between species (S2B Fig). For instance, the insertion domain of *Eimeria* and *Neospora* are very divergent whereas *Hh*DegP and *Tg*DegP share the most conserved insertion domain. We do not know the role of this domain and if it may impact the immune-regulatory function of *Tg*DegP, but it is temptative to speculate that this insertion reflects a species’ functional adaptation for the host infected. So far, studies focusing on strain-dependent virulence factors have highlighted that the difference of virulence observed between strains is mainly due to single nucleotide polymorphism (SNP) or changes in the level of expression. By comparing the cDNA of TgDegP between type I (RH) and type II (Pru) we found that DegP harbors a high degree of conservation between strains with the protease and PDZ domains that remain strictly identical while SNP can be only found in the sequence encoding for signal peptide and in the insertion region of TgDegP. However, it remains possible that SNPs present in the insertion domain contributes to the strain-dependent function of DegP. Another hypothesis is that the high degree of virulence of type I strain maskes the *in vivo* phenotype of KO-*DegP*^*I*^ in mice whereas the moderate virulence of the type II strain allows the visualization of this phenotype.

*Pf*DegP is the closest trpysin-like protease found in *P*. *falciparum*; despite a predicted signal peptide, many features show that they are very divergent. First, they are not associated with the same compartment. While, *Toxoplasma* DegP is a rhoptry bulb protein, immunolocalization of *Pf*DegP in the asexual blood stages of malaria parasite does not indicated an association with rhoptry but rather that the protease is synthetized during trophozoite stages (26-30h; before rhoptry biogenesis) and appears to be transported to the cytosol and membrane surface of infected RBCs during the late developmental stages of the parasite. Second, we showed that *Toxoplasma* DegP is subject to proteolytic cleavage that likely takes place in the rhoptry based on precedent for other rhoptry proteins. Proteolytic maturation of DegP proteases is unconventional, and seems to not undergo in the *P*. *falciparum* orthologue [39]. Finally, the insertion domain between the trypsin and PDZ domains does not exist in PfDegP (S2A Fig). Interestingly, this extension does not exist either in the duplicated *Toxoplasm*a DegP-like gene *TGME49_290840*, supporting the idea that *Pf*DegP might harbors a more similar architecture with TGME49_290840 than with *Tg*DegP. In addition, the cell cycle time course transcription of *TGME49_29084*0 does not show the typical periodicity of rhoptry proteins (ToxoDB.org) [29], dismissing a potential rhoptry localization. These observations reflect that the two closest DegP-like proteins of *Toxoplasma* have probably different locations and then functions, highlighting the versatility of roles played by this protease family.

## Supporting information

S1 Fig Generation of a DegP knock-out strain in PruΔ*Ku80* strain (KO-*DegP*^*II*^). (A) Strategy and (B) PCR verification of the correct integration of the vector by single homologous recombination at the endogenous *DegP* locus in PruΔ*Ku80* strain. The primers ML673 and ML871 are used in this PCR. The recombined locus was detectable only in transgenic parasites KO-*DegP*^*II*^, as shown by a specific amplification of a 707 bp fragment that is not amplified in the parental strain.
(TIF)

S2 Fig Alignment of the DegP homologs found in other Apicomplexa or Coccidia. (A) Local alignments generated between *Tg*DegP and homologs found in Apicomplexa: *Tg*DegP (*T*. *gondii* TGME49_262920), *Hh*DegP (*Hammondia hammondi* HHA_262920), *Nc*DegP (*Neospora caninum* NCLIV_025000), *Et*DegP (*Eimeria tenella* ETH_00028355), *Pf*DegP (*P*. *falciparum* PF3D7_0807700), *Pv*DegP (*P*. *vivax* PVX_088155), *Pcyn*DegP (*P*. *cynomolgi* PCYB_011950), *Tp*DegP (*Theileria parva* TP01_0318), *Bb*DegP (*Babesia bovis* BBOV_IV004330). (B) Local alignments generated between *Tg*DegP and its closest homologs found in Coccidia *Eimeria tenella*, *Neospora caninum* and *Hammondia hammondi*. Identical amino acids are highlighted in black and similar amino acids are shaded in grey. The catalytic domain and the two PDZ domains are highly conserved among Coccidia whereas the central region of the protein is the most divergent part.
(PDF)

## Abbreviations

DegP: Deg-like serine protein
HtrA: High temperature requirement A
Pru: Prugniaud
*Tg*: *Toxoplasma gondii*
