# Supplementary figures and images for: Characterization of Toxoplasma DegP, a rhoptry serine protease crucial for lethal infection in mice
Source: PLoS One. 2017 Dec 15;12(12):e0189556. doi: 10.1371/journal.pone.0189556 (PMC5731766; doi:10.1371/journal.pone.0189556)

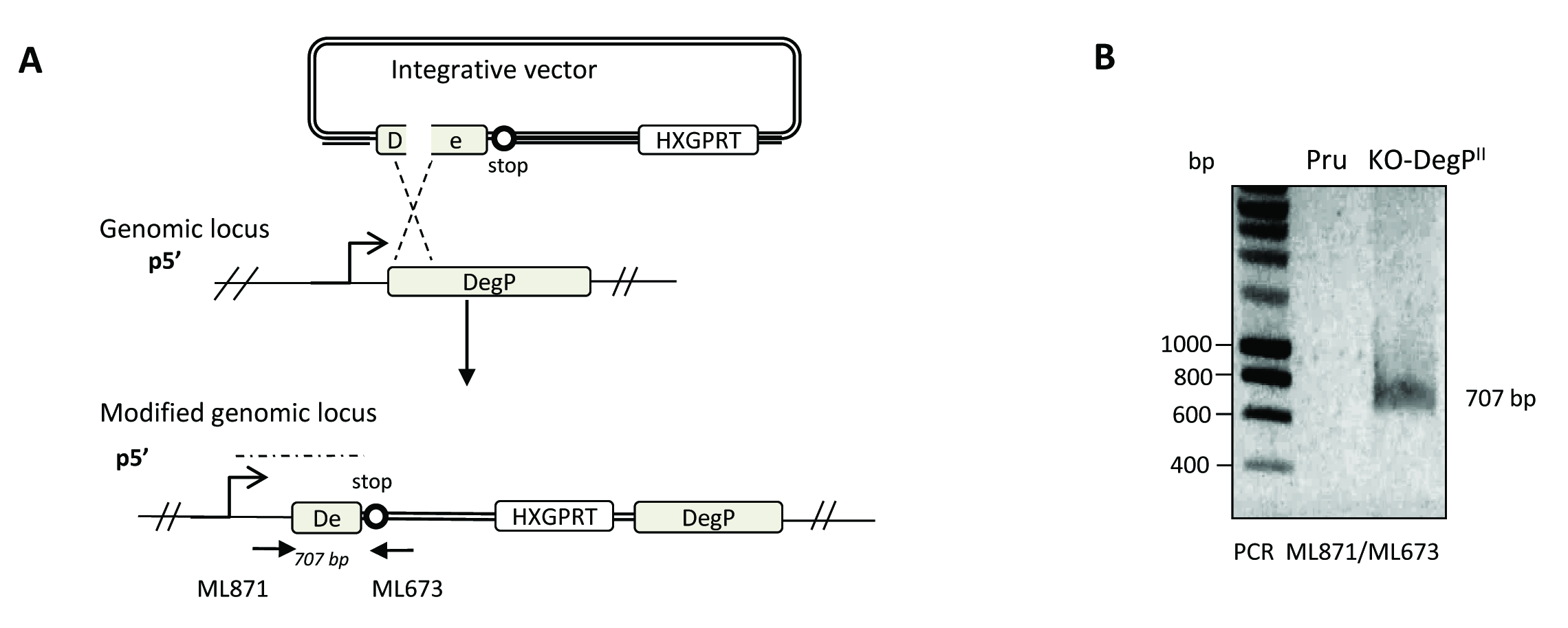

Supplement: S1 Fig — (A) Strategy and (B) PCR verification of the correct integration of the vector by single homologous recombination at the endogenous DegP locus in PruΔKu80 strain. The primers ML673 and ML871 are used in this PCR. The recombined locus was detectable only in transgenic parasites KO-DegPII, as shown by a specific amplification of a 707 bp fragment that is not amplified in the parental strain. (TIF) [file pone.0189556.s001.tif]
