# Supplementary material for: Characterization of Toxoplasma DegP, a rhoptry serine protease crucial for lethal infection in mice
Source: PLoS One. 2017 Dec 15;12(12):e0189556. doi: 10.1371/journal.pone.0189556 (PMC5731766; doi:10.1371/journal.pone.0189556)
Supplement: S2 Fig — (A) Local alignments generated between TgDegP and homologs found in Apicomplexa: TgDegP (T. gondii TGME49_262920), HhDegP (Hammondia hammondi HHA_262920), NcDegP (Neospora caninum NCLIV_025000), EtDegP (Eimeria tenella ETH_00028355), PfDegP (P. falciparum PF3D7_0807700), PvDegP (P. vivax PVX_088155), PcynDegP (P. cynomolgi PCYB_011950), TpDegP (Theileria parva TP01_0318), BbDegP (Babesia bovis BBOV_IV004330). (B) Local alignments generated between TgDegP and its closest homologs found in Coccidia Eimeria tenella, Neospora caninum and Hammondia hammondi. Identical amino acids are highlighted in black and similar amino acids are shaded in grey. The catalytic domain and the two PDZ domains are highly conserved among Coccidia whereas the central region of the protein is the most divergent part. (PDF) [file pone.0189556.s002.pdf]

A

```
PfDegP      1 MDIIFCTPTYCKIMLMIIMLI-------SLRTRCDTNNF-------------------LN
PvDegP      1 MKLSLQSYSCCAAVAALVVIRGGGDPWGGFFPRCAAHQMGERPPPTDERNYAENNGGKLG
PcynDegP    1 ------------------------------------------------------------
EtDegP      1 ------------------------------------------------------------
TpDegP      1 ------------------------------------------------------------
BbDegP      1 ------------------------------------------------------------
NcDegP      1 -------------MMAEQRDRSQRARWTAR------------------------------
TgDegP      1 ------------------------------------------------------------
HhDegP      1 ------------------MAALQRSRWPRAR------------------------TG---

PfDegP     35 CSVEKEEGEEEIISTNLKRINDDM-------------------------NILGRILNDER
PvDegP     61 GEIEKEEEEHLHIDREVKGDDSHFTFDESSPTGGGKNVKVGLPLAKYTPGYVRPVLSDKR
PcynDegP    1 ------------------------------------------------------------
EtDegP      1 ------------------------------------------------------------
TpDegP      1 ------------------------------------------------------------
BbDegP      1 ------------------------------------------------------------
NcDegP     18 -----------------------ALF-------LLAWLAVGLEMLFWNSNSSFVCPKAHA
TgDegP      1 ------------------------ML-------LLLSLFVALEVSSWISSDSVVSLKAHG
HhDegP     16 -----------------------MVL-------LLLSVFVALEVPSWIASYSVVPLKAHG

PfDegP     70 ---------------NIKITDI-------------------VEM----LQNEYDDKKKKK
PvDegP    121 VTKGEAPG----GENNLPSGDPQDGPPYLSKQQCGASFRDNAKADEHPSEGSHHDGEEPP
PcynDegP    1 ------------------------------------------------------------
EtDegP      1 ------------------------------------------------------------
TpDegP      1 ------------------------------------------------------------
BbDegP      1 ------------------------------------------------------------
NcDegP     48 VEVSSSSAERSFSPASTHSPGPRDAAPKLEA---------------------ASDGQTPL
TgDegP     30 AEASSLLT-----------PEPS-------------------------------------
HhDegP     46 AETSSLLT-----------PEPS-------------------------------------

PfDegP     92 KKKKYIYRKKKSNNKINEINSVQHFNVKENILNEKK-----KNPLNDNFKNPKLRKHSPN
PvDegP    177 SAAPNIWRRKKKNILQHRMSPTERGQEGYPPVEEKQTHREASNSDGSNLHESNFRGG-SS
PcynDegP    1 ------------------MNPTEHHLMDYSLMKDKQIHRGETLNS----YVNNIDDH-NS
EtDegP      1 ------------------------------------------------------------
TpDegP      1 ------------------------------------------------------------
BbDegP      1 ------------------------------------------------------------
NcDegP     87 AS-DTLFLDPST-TSTDEGSAEERF----QELEEDEFEHS---TSG-------TAQHSPR
TgDegP     42 -----------R------------------DLRE--------------------------
HhDegP     58 -----------R------------------DLRE--------------------------

PfDegP    147 NKKNKNKIGQ-IKFHIVGYDKKKITKYLTPSMISSIQKRLMNKNKKTN------------
PvDegP    236 YTEGSHQVGQ-LKLRTNRDDQKMMKGIFTPQVVRAMKEYLLKGGKKYGSGQVGHLGGSSD
PcynDegP   38 KRRMQPRVGK-LKLRINRDDKKTIRDTLTPQVVKVMKEYLLKGRKKYRNGQMDHLGGISS
EtDegP      1 -------------MRIILALSLSLTATFVDS-------YLLVTNEVGN----GTPASS--
TpDegP      1 ------------------------------------------------------------
BbDegP      1 ------------------------------------------------------------
NcDegP    131 EDIAVPQDGSESPFTAFISESEPGEESFEPS-------T-RIPREPTE----ETPAAFSF
TgDegP     47 DATVLHEDGFVSTFPAAVATSAHGAEPLPPE-------R-EITEEVTG----EMLVGSSL
HhDegP     63 DAAVLHEDGFVSTFPAAVATSAQVAEQLPTE-------R-EITEEVAG----EMLVGSSL
```

```
PfDegP     194 -----------------------VNVNMSNGRVLPFFPKEYFLHSSTHEKKDKMATKKNN
PvDegP     295 QQHGGDRRTGSNQAIHPLSGEAIHPLSSEAIHPLRFFPANFAPHSGDSQ--------RGA
PcynDegP    97 DQLDRRDGTGWGSS---------WSGSSKVNRVLRFFPANFAPHSGDNQ--------RGA
EtDegP      35 ------ASTGNGG-----------------------------------------VSLGFKD
TpDegP       1 ------------------------------------------------------------
BbDegP       1 ----------------------------------------------------------MA
NcDegP     179 LQVNAAAALAKGQ-----------------------------------------ISPSRIE
TgDegP      95 LQVSAAGQP-------------------------------------------------TE
HhDegP     111 LQVSAAGQP-------------------------------------------------ME

PfDegP     231 KEESIINERLLEN---------LENISLSRTIKDIRGEE-----------GNER------
PvDegP     347 ARRSIINEKLRRN---------LSQITLSVSAAEERTKE-------RVEEGAEVGAQ---
PcynDegP   140 AKRSIINEKLHRN---------LKQITLGAAGSGDADGE-------RAEDAKK-GAKDAK
EtDegP      49 APTGAIQGGLHE--ANPSAFQ---EFSLIEEGAPIESKTGSVNEAERQKSTPEGGYLSA-
TpDegP       1 ------------------------------------------------------------
BbDegP       3 YSTSALYVRLLSQNVKPAAS------CYTVFGTGNC-FHH-AKVHKHLASETD--A----
NcDegP     199 LAKGLAAAALAKGQISPSRIELAKGLAAAALAKGQISPSR-IELAKGLAAAPA--APAQ-
TgDegP     106 LAA--------------------------AAAEGVNSQGG-NTLAKTLAAAAA----SQ-
HhDegP     122 LAA--------------------------AAGENVNSQGG-NVLAETLAAAAA----SQ-

PfDegP     265 ----KVKEEDNNIKEEGNSFKKYFKGVVKLYVDITEPNLEMIWQNYPPKSITGSGFIIEG
PvDegP     388 ----SVAQSGGADDGEAGALRKIYKGVVKLYVDITEPSLETIWSNSPPKRVSGSGFVIEG
PcynDegP   183 DAKDIKDARDAKNNAEAGALHNIYRGVVKLYVDITEPSLEMIWSNSPPKRVTGSGFVIEG
EtDegP     103 -----KRKSTQAKSSVNLTEVEEFASVVKIFVDAVKADFVSPWQMMAPKEQTGSGFVVEG
TpDegP       1 ------------------------------------------------------------
BbDegP      49 -----SHDLQFYDYRSHQLLKRSFGSIVKIYCDSTDPNYAQPWQMRRQLKSIGSGFAISN
NcDegP     255 -----KETATQGTRAGRHLLSSTLSSVVKIFVDFTLPDYYSPWQMQAPKEASGSGFVVEG
TgDegP     134 -----KQ---TTAKDRHALLTSSLSSVVKIFVDITMPDYFSPWQMQAPKDASGSGFVVEG
HhDegP     150 -----KQ---TTAKDRHALLTSTLSSVVKIFVDITMPDYFSPWQMQSPKDASGSGFVVEG

PfDegP     321 HLIIITNAHNISYSTRILIRKHGNSGKYEAKILYVAHDVDIAILTTDDKTFFDDVYALHFG
PvDegP     444 GLILTNAHNVAYSTRILVRKHGCSKKYEGAVLHVAHEADMALLTVADGSFYEDVSALELG
PcynDegP   243 DLILTNAHNVAYSTRILVRKHGSSKKYEATVLHVAHEADIAILTVSDRSFYQDVSALELG
EtDegP     158 RMIMTNAHLIADQTRVLVRRHGNPKRFLARVLAVCHECDLALVTVDDDVFWERIKPLAFG
TpDegP       1 ---------------MLMVWCRSTRKYIARMIEIGHECDLAVLTVDDESFWDGITPLEFG
BbDegP     104 RMVLTNAHCVSWHNRCLLRKHGSTIKFPGRIVAIGHECDLAIIHVDSEEFWEGVEPLELG
NcDegP     310 KRILTNGHVVAETTRVLVRKHGNAKKFLARVLATAHEADLALLEVDSEEFWENLQPLPFG
TgDegP     186 KRILTNGHVVGETTRVLVRKHGNAKKFLARVVATAHEADLALLEVESDEFWENLQPLPFG
HhDegP     202 KRILTNGHVVGETTRVLVRKHGNAKKFLARVVATAHEADLALLEVESGEFWENLQPLPFG

PfDegP     381 ALPSLKDEIITIGYPAGGDKLSVTEGIVSRIDVQYYKHSNYK------------------
PvDegP     504 PLPSLRDDVITVGYPSGGDKLSVTKGIVSRIEVQYYRHSNSR------------------
PcynDegP   303 PLPSLRDDVITVGYPSGGDKLSVTKGIVSRIEVQYYKHSNER------------------
EtDegP     218 GVPQLRETVVVLGYPTGGDQLSITEGVVSRVGVSMYAHSSLG------------------
TpDegP      46 DVPNLHDNVTVIGYPTGGDNLCITSGVVSRVDVTTYSHSNFRYLTSLHYLHIFYYYTNVS
BbDegP     164 EVPSLHDAVTVVGYPAGGDNLCITSGVVSRVDVTTYAHSNFR------------------
NcDegP     370 GIPRLRDSVTVLGYPTGGDQLSITEGIVSRVGMSAYAHSSVS------------------
TgDegP     246 GIPRLRDSVTVLGYPTGGDQLSITEGIVSRVGMSMYAHSSVS------------------
HhDegP     262 GIPLLRDSVTVLGYPTGGDQLSITEGIVSRVGMSMYAHSSVS------------------
```

```
PfDegP     423 ----------FLLTQIDAPLNPGNSGGPALVRGKVVGICFQSYKVSNNISYIIPSTIISH
PvDegP     546 ----------LLLTQIDAPMNPGNSGGPALVKGKVAGICFQLLKMANNTSYIIPTPVIKH
PcynDegP   345 ----------LLLTQIDAPMNPGNSGGPALVKGKVAGICSQLLKTANNTSYIIPTPVIKH
EtDegP     260 ----------LLTVQIDAPINPGNSGGPALAAGKVVGVAFQGFSEMQNVGYIVPFPVIRH
TpDegP     106 FYTFLHTNLQLLCVQIDAAINSGNSGGPAIKDGRVIGVAFQAYDEAQNIGYIIPTCIINQ
BbDegP     206 ----------LLCAQIDAAINAGNSGGPALKDGKVIGVAFQAYDEAQNIGYIIPTSIVRQ
NcDegP     412 ----------LLTVQIDAAINPGNSGGPAVVDGRVVGVAFQGFSQLQNVGYIVPYPIVRH
TgDegP     288 ----------LLTVQIDAAINPGNSGGPALVDGRVVGVAFQGFSHLQNVGYIVPYPIIEH
HhDegP     304 ----------LLTVQIDAAINPGNSGGPALVDGRVVGVAFQGFSHLQNVGYIVPYPIIEH

PfDegP     473 FLLDIHKNKDYTGYAFLGVKYEPLENPSLREALGLEEMERKKII--KKNVGILITEVFEG
PvDegP     596 FLMDLHRSGKYNGYPSLGVKYLPLDNANLRRLLGLTDLERRREV--EENSGILVTEVDEE
PcynDegP   395 FLLDLHKSGKYNGYPSLGVKYVPLDNANLRRLVGLTDLEKGKAV--EENSGILVTEVDEE
EtDegP     310 FLNDIALHKQHTGIVSLGIKAQPMENEALKRFKGMDTLPPEALPENVTASGVLVVSVDKV
TpDegP     166 FLKQIQLFNKYTGFVNIGITYQLLTNPFLKSFLSHKQHNTEL----GLGGGIMVCQYDES
BbDegP     256 FLRQLEIHNRYTGFVTIGITYQLLENPALRSFVGLDQINASELPEGITATGILVCQCDKV
NcDegP     462 FLNDLVLHGRYTGFPSLGVKIAHMENDHLRQFKGLSALTAADLPPGVTPTGVLVVEVDNL
TgDegP     338 FLNDLVLHGRYTGFPSLGVKVSHMENDHLRQFKGLSALKASDLPPGVTPTGVLVVEVDNL
HhDegP     354 FLNDLVLHGRYTGFPSLGVKVSHMENDQLRQFKGLSALKASDLPPGVTPTGVLVVEVDNL

PfDegP     531 HMSKQDDKYHD------------------M------------------------------
PvDegP     654 QMGCQSGGGGS------------------T------------------------------
PcynDegP   453 QMRYQPADVDY------------------C------------------------------
EtDegP     370 RRTIYTQGKIALPLSRYSQIPESSSAGKRCVRYKAHVGPSIAAFDSSMAKCELMKPILLV
TpDegP     222 L-----------------------------------------------------------
BbDegP     316 P-----------------------------------------------------------
NcDegP     522 RVSRYKTGQIRVPYASRSLAEPRD-------------------------RLELLQELRAQ
TgDegP     398 RVSRYKAGKIRVPYTSRTLSGPRN-------------------------L-KMMQSVQAQ
HhDegP     414 RVSRYKAGKIRVPYVSRTLSGPRN-------------------------L-KMMQRVEVQ

PfDegP     543 -----------------------------DNKHHNVG-----------------------
PvDegP     666 --------------------------------KWAVS-----------------------
PcynDegP   465 --------------------------------AYAEG-----------------------
EtDegP     430 D-QPVPR--ENDTTEHQRNVKEIAARVIWQPENWKPSTAASCSYCHHQQLIPREKRNVLQ
TpDegP     223 ------------------------------------------------------------
BbDegP     317 ------------------------------------------------------------
NcDegP     557 DEESAPPAAEARTSELGAAE--SG-GQMDAPRFELVSA----------------------
TgDegP     432 EDVSLPSATSSG---HGASF--S--GSLASPVPDFVKEA-----APPGQMLSAPLGGETP
HhDegP     448 EDASLPSVTSSG---HGASF--S--GSLASPVPDIVKEA-----APSGQMLSAPLRGETP

PfDegP     551 -------------------DTHHNVGD---------------------------------
PvDegP     671 -------------------SGEASTGA---------------------------------
PcynDegP   470 -------------------ATQA-------------------------------------
EtDegP     487 NQKKVSQSGTPEA-----ADVQRETGPMQWVKTIVQVEDVKGVADAEGSRLLSTVETKEK
TpDegP     223 ------------------------------------------------------------
BbDegP     317 ------------------------------------------------------------
NcDegP     592 ---ASAASGDVQS------LAHREADGGLAHPAFLQTRITPTHILGSRSQLLRLYALAR-
TgDegP     480 ESAVVAAGGGEKPAARAFGQGGSEAGPSRSQPTFLQTQVTPKHILANRSQLLRLYALAR-
HhDegP     496 ESAVVATGGGEKPAARAFGQGGSETGPSRSQPTFLQTQVIPKHIRASRSQLLRLYALAR-
```

```
PfDegP    559 ----------TH-----------------------------------------HNVG---
PvDegP    679 ----------ASS--------------------------------------GEASMG---
PcynDegP  474 ------------------------------------------------------------
EtDegP    542 HLRRVSAQGSTSNREVNEESAPQSGQDARMASPLLRADIESAPQSGQDAKTASPQLGADT
TpDegP    223 ------------------------------------------------------------
BbDegP    317 ------------------------------------------------------------
NcDegP    642 --RR------QQVHEVTE-----------------------------GA--ASPSEGREL
TgDegP    539 --RR------QLTREAVA-----------------------------DEAAADSGEERGL
HhDegP    555 --RR------QLAREAVA-----------------------------DEAAADSGEERGP

PfDegP    565 ------------------------------------------------------------
PvDegP    688 ------------------------------------------------------------
PcynDegP  474 ------------------------------------------------------------
EtDegP    602 AGVHESTSARSEPATLDLGVRKKAESAGALKYTPTNSSGHGSGDEDDERQKGQGRPDLRT
TpDegP    223 ------------------------------------------------------------
BbDegP    317 ------------------------------------------------------------
NcDegP    663 VATGHGDRSTPPPIVVNMTVAKLG--PGLYAQIPKG--AFG----PPVIPVSRRLPGIRD
TgDegP    562 VETQGARTTAP-PVMVRMVVRKLG--PGIYSQIPEG--DRS----FRARE-GSGFP--KP
HhDegP    578 VETQGARTTAPPPAAVNMVVRKLG--PGIYSQIPEG--GGS----FRARQGGSGFP--KP

PfDegP    565 --------------------------------------------------DDHTDNL-QG
PvDegP    688 --------------------------------------------------AASTGAASSG
PcynDegP  474 ------------------------------------------------------------
EtDegP    662 QTGQTPVPKKRRSHTSNIRASLESLLREILRGSSKLRQVRNILLGR--GGGDESDNLRGT
TpDegP    223 ------------------------------------------------------------
BbDegP    317 ------------------------------------------------------------
NcDegP    715 WRL--------------------------------QSAPDSAARGENPGVAKDTGHVLEA
TgDegP    610 WSA----------------------FRMLQRR---LRNVQVTARGEKTGGEDETGDLSQD
HhDegP    628 WYA----------------------FRMLQRR---LRNAQMTARGEKAGGEGETGDLSQD

PfDegP    574 DTDY-----CTYILNSNIIT------------------------------SDKKNIYSDK
PvDegP    698 EASMG---AASTGAASHAIT------------------------------AGRGP-L---
PcynDegP  474 EVSTG---Q-ASSTGAHALA------------------------------TGVDP-F---
EtDegP    720 FSATNTKRKAEQASNSNDNTQ--NEDALEKRPVVSGLKLAGDNTAGSAESNDSSPRSSNT
TpDegP    223 ------------------------------------------------------------
BbDegP    317 ------------------------------------------------------------
NcDegP    743 QEHAQDQAHDEVASGRNAL-------EVQENRVVRG-NARGALPNGS--P---SPTSSEA
TgDegP    645 QEAKGNREEPEQVEGKVLVSPENVPGSIQKTGALPV-NMRGPLLSTA--LPGSSSISSFP
HhDegP    663 QEAEGDRKEPEQVEGKALVLPENVPGSVQKTGVLPV-NMRGPLPPRA--LSDPSSVSSLP

PfDegP    599 KKK------------------------KIYS-------------DNNNNNNNFNYYYNMH
PvDegP    721 ---------------------------------------------------PGRPPITPT
PcynDegP  496 ---------------------------------------------------PRRPP----
EtDegP    778 SSKELRSEERTATENDAEEKRNLEEKSQKYESHDNERETEDTALAVAVHPLVDNPYFNPA
TpDegP    223 --------------------------------------------R---------------
BbDegP    317 --------------------------------------------R---------------
NcDegP    790 PNQQTA---RKA------------------------VEPLEEGPRSEEELLLPNPYFPPE
TgDegP    702 AQEPLS---EKA------------------------SEAVEEGPRSEEELLLPNPYFHQ-
HhDegP    720 AQDLLS---GKA------------------------GEAIEEGPRSEEELLLPNPYFHQ-
```

```
PfDegP     622 GEDEQSCYGLKKNDIILRVDGKDINNDGSVILRDNETVGFQHLFNEKFINDLCIIKIVRN
PvDegP     730 APAEPLCYGLKKNDVLLSVEGTQIKSDGTVTLRGDESVDFQFLFNGKFVGDLCTCRVVRG
PcynDegP   501 --TDAHCYGLKKNDVIISIDGTQIKSDGTATLRGDESVDFQFLLNEKYVQDLCTCRVVRK
EtDegP     838 FLDEKEFLGFQEGDVILSIGNYS---------MDLERVSFHYVITQYFNGETTWAYVLRD
TpDegP     224 -------GVIETNDVILQINGHPIADDGTVHFRGDERVHLGYSLTDKFCGDDCELLILRN
BbDegP     318 -----PGDRLQTRDVILAINGHDVADDGTVHFRDVERVHLAYALTNKFTGDPCEVVVLRD
NcDegP     823 QDVEGGELGLKVGDVILAVDGTDVADDGTVAFRQLERVSIDYTIMNRFNGETCKVLVLRD
TgDegP     734 QDAEENEIGFKVGDVILAIDGIDVADDGTVAFRQLERVSIDYTIMKRFNGETCKALVLRD
HhDegP     752 QDAEENEIGFKVGDVVLAIDGIDVADDGTVAFRQLERVSIDYTIMKRFNGETCKALVLRD

PfDegP     682 KKIKSVMVKLYKVKYLLNQHNWDKRNKYFIYGGIVFSILTRSLYVYT--QNPEI--NKLM
PvDegP     790 GRVQTALVRVSRVHYLVRQHNWDVPNKFFIYGGVVFTTLTRSLYADEETDNVEV--MRLL
PcynDegP   559 GRIKSVVVRVSRVNYLVRQHNRDVRNKFFIYGGVIFTTLTRSLYPDEDTDNVEL--LRLL
EtDegP     889 NRVIKITVPLMTPNFKVPPFTWDMKPSYFVYGGFVFTPLSKVLLATKLRKEAPHMFGFLV
TpDegP     277 NKLKKIHIKLGKPSYLVPEHQWDIMPRYYIYAGLVFIPLSMEYLKD----EFGKKFYERA
BbDegP     373 GVVKTLVVHLQRPSYLVPEHQWEVMPRYYIFGGLVFVPLTMEYLKD----EFGKKFYERA
NcDegP     883 GRVREILVPITNLNLKIPRHTWDQKPKYFVFGGLVFTTLTRQLLEHMKAAEFPAHFYTKI
TgDegP     794 GQVREVLLPITNLNLKVPAHTWDQKPKYFVFGGLVFTTLTRHLLEHMKLTEFPAEFFTKI
HhDegP     812 GQVREVLLPITNMNLKVPTHTWDQKPKYFVFGGLVFTTLTRHLLEHMKLTEFPTEFFNKI

PfDegP     738 L-------YNNFKKKSKDEIVVLKNILPTKITTGYYYTDSIVLRVNNIKVKNLKHLIELI
PvDegP     848 Q-------FNLFKKQRDDEIVIVKRILPSKLTIGFNYQDCIVLTVNGIPVRNLQHLVGVI
PcynDegP   617 Q-------FNLFKKRRGDEIVIVKRILPSKLTIGFNYTDCIVLTVNGIPVRSLQHLVEVI
EtDegP     949 Q-------RLDYQEEAGDEFVVLSLILASDISVGYEVPPCIVHSVNGHHVRNMQDLVRFI
TpDegP     333 PTALLKPLSDMFARESGEEVVILSQILASDLTIGYDFKNIRLVSVNNTKILNLRHLEQVL
BbDegP     429 PSALLKPLSDIFADESGQEVVVLSQILASDITVGYDFRNVRLEAVNGHSVRNLKHLEYLL
NcDegP     943 K-------RSEYQEAEGDEVVVLSVILASELTVGYNAAPAIVTAVQGQKVRGLADVVRIV
TgDegP     854 K-------QTKYQEEEGDEVVVLSVILASELTVGYTAAPAIVTAVQGQKVRGLADVVRIV
HhDegP     872 K-------KAKYQEEEGDEVVVLSVILASELTVGYTAAPAFVTAVQGEKVRGLAEVVRIV

PfDegP     791 EMTKYTNRL-----------MYLKNN---------------KHTLQSYINYYNTK----I
PvDegP     901 DGREGPLGGGVAKKSTQMKQM---------DQMGERRGTPSEGDANPYLKFTLEEDPFFQ
PcynDegP   670 DKRDGPAGGEAANMANAANMANAANTANTASTANTKEVPSTSGNANPYLMSTLENDPFLH
EtDegP    1002 EAKD--G-----------------------------------------------------
TpDegP     393 LSAV--E-----------------------------------------------------
BbDegP     489 QNTLRDS-----------------------------------------------------
NcDegP     996 EESK--E-----------------------------------------------------
TgDegP     907 EQST--D-----------------------------------------------------
HhDegP     925 EQST--D-----------------------------------------------------

PfDegP     821 TSLNINTIIHILILTTSGQKVPIVLNKRDVEKYNEEIKKIYSITRDRYVY---------
PvDegP     952 MKEEKDNLLHFLLLTSNGQQVPLVLLRAEVQRESAEIRRVYGITRERYVYSG-------
PcynDegP   730 VQ-EKDNLLHFLLLTSNGQQVPLVLLRNEVQQESAEIKRVYGITRERYLYNG-------
EtDegP    1007 ------DFVELQLEANEADKLHVAIDRKKAAIQQKVLKDHNIVSDRSPDLIRKKINSL
TpDegP     398 -------KYVKFQFE----QDILVVLETAKVPEFEQQILQQHAISAHKSRII---------
BbDegP     496 -------PYLTFKFD----GEVILVLDRIKAEELHPLILEQHAIPAHKSREL---------
NcDegP    1001 ------NFLEFTVKVSGISQMPIVLDRQKAMAVNPKILGQHKILRDRSFFL---------
TgDegP     912 ------NFLEFTVKISGISALPIVLDRKKAMAVNPKILGQHKILRDRSYFL---------
HhDegP     930 ------NFLEFTVKISGISQLPIVLDRKKAMAVNPKILGQHKILRDRSYFL---------
```

B

```
EtDegP     1 ------------------------------------------------------------
NcDegP     1 MMAEQRDRSQRARWTAR---ALFLLAWLAVGLEMLFWNSNSSFVCPKAHAVEVSSSSAER
TgDegP     1 ---------------------MLLLLSLFVALEVSSWISSDSVVSLKAHGAEASSLLT--
HhDegP     1 -----MAALQRSRWPRARTGMVLLLLSVFVALEVPSWIASYSVVPLKAHGAETSSLLT--

EtDegP     1 ------------------------------------------------------------
NcDegP    58 SFSPASTHSPGPRDAAPKLEAASDGQTPLASDTLFLDPSTTSTDEGSAEERFQELEEDEF
TgDegP    38 ---------PEPS--------------------------R-------------DLRE---
HhDegP    54 ---------PEPS--------------------------R-------------DLRE---

EtDegP     1 --------------------------MRIILALSLSLTATFVDSYLLVTNEVGNGTPASS
NcDegP   118 EHSTSGTAQHSPREDIAVPQDGSESPFTAFISESEPGEESFEPST-RIPREPTEETPAAF
TgDegP    47 -------------DATVLHEDGFVSTFPAAVATSAHGAEPLPPER-EITEEVTGEMLVGS
HhDegP    63 -------------DAAVLHEDGFVSTFPAAVATSAQVAEQLPTER-EITEEVAGEMLVGS

EtDegP    35 --------ASTGNGGVSLGFKDAPTGAIQGGLHE--ANPSAFQE---FSLIEEGAPIESK
NcDegP   177 SFLQVNAAAALAKGQISPSRIELAKGLAAAALAKGQISPSRIELAKGLAAAALAKGQISP
TgDegP    93 SLLQVSAAGQP---------TELAA--------------------------AAAEGVNSQ
HhDegP   109 SLLQVSAAGQP---------MELAA--------------------------AAGENVNSQ

EtDegP    82 TGSVNEAERQKSTPEGGYLSAKRKSTQAKSSVNLTEVEEFASVVKIFVDAVKADFVSPWQ
NcDegP   237 SR-IELAKGLAAAPA--APAQKETATQGTRAGRHLLSSTLSSVVKIFVDFTLPDYYSPWQ
TgDegP   118 GG-NTLAKTLAAAAA----SQKQ---TTAKDRHALLTSSLSSVVKIFVDITMPDYFSPWQ
HhDegP   134 GG-NVLAETLAAAAA----SQKQ---TTAKDRHALLTSTLSSVVKIFVDITMPDYFSPWQ

EtDegP   142 MMAPKEQTGSGFVVEGRMIMTNAHLIADQTRVLVRRHGNPKRFLARVLAVCHECDLALVT
NcDegP   294 MQAPKEASGSGFVVEGKRILTNGHVVAETTRVLVRKHGNAKKFLARVLATAHEADLALLE
TgDegP   170 MQAPKDASGSGFVVEGKRILTNGHVVGETTRVLVRKHGNAKKFLARVVATAHEADLALLE
HhDegP   186 MQSPKDASGSGFVVEGKRILTNGHVVGETTRVLVRKHGNAKKFLARVVATAHEADLALLE

EtDegP   202 VDDDVFWERIKPLAFGGVPQLRETVVVLGYPTGGDQLSITEGVVSRVGVSMYAHSSLGLL
NcDegP   354 VDSEEFWENLQPLPFGGIPRLRDSVTVLGYPTGGDQLSITEGIVSRVGMSAYAHSSVSLL
TgDegP   230 VESDEFWENLQPLPFGGIPRLRDSVTVLGYPTGGDQLSITEGIVSRVGMSMYAHSSVSLL
HhDegP   246 VESGEFWENLQPLPFGGIPLLRDSVTVLGYPTGGDQLSITEGIVSRVGMSMYAHSSVSLL

EtDegP   262 TVQIDAPINPGNSGGPALAAGKVVGVAFQGFSEMQNVGYIVPFPVIRHFLNDIALHKQHT
NcDegP   414 TVQIDAAINPGNSGGPAVVDGRVVGVAFQGFSQLQNVGYIVPYPIVRHFLNDLVLHGRYT
TgDegP   290 TVQIDAAINPGNSGGPALVDGRVVGVAFQGFSHLQNVGYIVPYPIIEHFLNDLVLHGRYT
HhDegP   306 TVQIDAAINPGNSGGPALVDGRVVGVAFQGFSHLQNVGYIVPYPIIEHFLNDLVLHGRYT

EtDegP   322 GIVSLGIKAQPMENEALKRFKGMDTLPPEALPENVTASGVLVVSVDKVRRTIYTQGKIAL
NcDegP   474 GFPSLGVKIAHMENDHLRQFKGLSALTAADLPPGVTPTGVLVVEVDNLRVSRYKTGQIRV
TgDegP   350 GFPSLGVKVSHMENDHLRQFKGLSALKASDLPPGVTPTGVLVVEVDNLRVSRYKAGKIRV
HhDegP   366 GFPSLGVKVSHMENDQLRQFKGLSALKASDLPPGVTPTGVLVVEVDNLRVSRYKAGKIRV

EtDegP   382 PLSRYSQIPESSSAGKRCVRYKAHVGPSIAAFDSSMAKCELMKPILLVD-QPVPR--END
NcDegP   534 PYASRSLAEPRDR-------------------------LELLQELRAQDEESAPPAAEAR
TgDegP   410 PYTSRTLSGPRNL--------------------------KMMQSVQAQEDVSLPSATSSG
HhDegP   426 PYVSRTLSGPRNL--------------------------KMMQRVEVQEDASLPSVTSSG
```

```
EtDegP  439 TTEHQRNVKEIAARVIWQPENWKPSTAASCSYCHHQQLIPREKRNVLQNQKKVSQSGTPE
NcDegP  569 TSELGAAES--G-GQMDAPRFELVSA-------------------------ASAASGDVQ
TgDegP  444 ---HGASFS----GSLASPVPDFVKEA-----APPGQMLSAPLGGETPESAVVAAGGGEK
HhDegP  460 ---HGASFS----GSLASPVPDIVKEA-----APSGQMLSAPLRGETPESAVVATGGGEK

EtDegP  499 A-----ADVQRETGPMQWVKTIVQVEDVKGVADAEGSRLLSTVETKEKHLRRVSAQGSTS
NcDegP  601 S------LAHREADGGLAHPAFLQTRITPTHILGSRSQLLRLYALAR---RR------QQ
TgDegP  492 PAARAFGQGGSEAGPSRSQPTFLQTQVTPKHILANRSQLLRLYALAR---RR------QL
HhDegP  508 PAARAFGQGGSETGPSRSQPTFLQTQVIPKHIRASRSQLLRLYALAR---RR------QL

EtDegP  554 NREVNEESAPQSGQDARMASPLLRADIESAPQSGQDAKTASPQLGADTAGVHESTSARSE
NcDegP  646 VHEVTE-----------------------------GA--ASPSEGRELVATGHGDRSTPP
TgDegP  543 TREAVA-----------------------------DEAAADSGEERGLVETQGARTTAP-
HhDegP  559 AREAVA-----------------------------DEAAADSGEERGPVETQGARTTAPP

EtDegP  614 PATLDLGVRKKAESAGALKYTPTNSSGHGSGDEDDERQKGQGRPDLRTQTGQTPVPKKRR
NcDegP  675 PIVVNMTVAKLG--PGLYAQIPKG--AFG----PPVIPVSRRLPGIRDWRL---------
TgDegP  573 PVMVRMVVRKLG--PGIYSQIPEG--DRS----FRARE-GSGFP--KPWSA---------
HhDegP  590 PAAVNMVVRKLG--PGIYSQIPEG--GGS----FRARQGGSGFP--KPWYA---------

EtDegP  674 SHTSNIRASLESLLREILRGSSKLRQVRNILLGR--GGGDESDNLRGTFSATNTKRKAEQ
NcDegP  718 -----------------------QSAPDSAARGENPGVAKDTGHVLEAQEHAQDQAHDEV
TgDegP  613 -------------FRMLQRR---LRNVQVTARGEKTGGEDETGDLSQDQEAKGNREEPEQ
HhDegP  631 -------------FRMLQRR---LRNAQMTARGEKAGGEGETGDLSQDQEAEGDRKEPEQ

EtDegP  732 ASNSNDNTQ--NEDALEKRPVVSGLKLAGDNTAGSAE---SNDSSPRSSNTSSKELRSEE
NcDegP  755 ASGRNAL-------EVQENRVV------RGNARGALPNGSP---SPTSSEAPNQQTA---
TgDegP  657 VEGKVLVSPENVPGSIQKTGAL------PVNMRGPLLSTALPGSSSISSFPAQEPLS---
HhDegP  675 VEGKALVLPENVPGSVQKTGVL------PVNMRGPLPPRALSDPSSVSSLPAQDLLS---

EtDegP  787 RTATENDAEEKRNLEEKSQKYESHDNERETEDTALAVAVHPLVDNPYFNPAFLDEKEFLG
NcDegP  796 RKA------------------------VEPLEEGPRSEEELLLPNPYFPPEQDVEGGELG
TgDegP  708 EKA------------------------SEAVEEGPRSEEELLLPNPYFHQ-QDAEENEIG
HhDegP  726 GKA------------------------GEAIEEGPRSEEELLLPNPYFHQ-QDAEENEIG

EtDegP  847 FQEGDVILSIGNYS---------MDLERVSFHYVITQYFNGETTWAYVLRDNRVIKITVP
NcDegP  832 LKVGDVILAVDGTDVADDGTVAFRQLERVSIDYTIMNRFNGETCKVLVLRDGRVREILVP
TgDegP  743 FKVGDVILAIDGIDVADDGTVAFRQLERVSIDYTIMKRFNGETCKALVLRDGQVREVLLP
HhDegP  761 FKVGDVVLAIDGIDVADDGTVAFRQLERVSIDYTIMKRFNGETCKALVLRDGQVREVLLP

EtDegP  898 LMTPNFKVPPFTWDMKPSYFVYGGFVFTPLSKVLLATKLRKEAPHMFGFLVQRLDYQEEA
NcDegP  892 ITNLNLKIPRHTWDQKPKYFVFGGLVFTTLTRQLLEHMKAAEFPAHFYTKIKRSEYQEAE
TgDegP  803 ITNLNLKVPAHTWDQKPKYFVFGGLVFTTLTRHLLEHMKLTEFPAEFFTKIKQTKYQEEE
HhDegP  821 ITNMNLKVPTHTWDQKPKYFVFGGLVFTTLTRHLLEHMKLTEFPTEFFNKIKKAKYQEEE
```

```
EtDegP  958 GDEFVVLSLILASDISVGYEVPPCIVHSVNGHHVRNMQDLVRFIEAKDGDFVELQLEANE
NcDegP  952 GDEVVVLSVILASELTVGYNAAPAIVTAVQGQKVRGLADVVRIVEESKENFLEFTVKVSG
TgDegP  863 GDEVVVLSVILASELTVGYTAAPAIVTAVQGQKVRGLADVVRIVEQSTDNFLEFTVKISG
HhDegP  881 GDEVVVLSVILASELTVGYTAAPAFVTAVQGEKVRGLAEVVRIVEQSTDNFLEFTVKISG

EtDegP 1018 ADKLHVAIDRKKAAAIQQKVLKDHNIVSDRSPDLIRKKINSL
NcDegP 1012 ISQMPIVLDRQKAMAVNPKILGQHKILRDRSFFL--------
TgDegP  923 ISALPIVLDRKKAMAVNPKILGQHKILRDRSYFL--------
HhDegP  941 ISQLPIVLDRKKAMAVNPKILGQHKILRDRSYFL--------
```
